# Can neuroscience help to understand narcissism? A systematic review of an emerging field

**DOI:** 10.1017/pen.2021.1

**Published:** 2021-05-28

**Authors:** Emanuel Jauk, Philipp Kanske

**Affiliations:** 1 Clinical Psychology and Behavioral Neuroscience, Faculty of Psychology, Technische Universität Dresden, Dresden, Germany; 2 Department of Psychology, University of Graz, Graz, Austria; 3 Max Planck Institute for Human Cognitive and Brain Sciences, Leipzig, Germany

**Keywords:** Grandiose narcissism, Vulnerable narcissism, Pathological narcissism, Narcissistic personality disorder, Personality functioning, Stress reactivity, Social affect, Social cognition

## Abstract

Narcissism is a Janusian personality construct, associated with both grandiose self-assuredness and dominance, as well as vulnerable insecurity and reactivity. Central questions of intra- and interpersonal functioning in narcissism are still a matter of debate. Neuroscience could help to understand the paradoxical patterns of experience and behavior beyond the limitations of self-reports. We provide a systematic review of 34 neuroscience studies on grandiose, vulnerable, pathological narcissism, and Narcissistic Personality Disorder (NPD), spanning experimental investigations of intra- and interpersonal mechanisms, research on neurophysiological and neuroendocrine aspects of baseline function, and brain structural correlates. While neuroscience has scarcely directly studied vulnerable narcissism, grandiose narcissism is associated with heightened vigilance to ego threat and stress responses following ego threat, as well as heightened stress indicators in baseline measures. Such responses are not commonly observed in self-reports, highlighting the potential of neuroscience to augment our understanding of self-regulatory dynamics in narcissism. Interpersonal functioning is characterized by deficits in social–affective processes. Both involve altered activity within the salience network, pointing to a double dissociation regarding the expression of narcissism and self/other oriented situational focus. Findings are summarized in an integrative model providing testable hypotheses for future research along with methodological recommendations.

## 1. Narcissism: Concepts and Operationalizations

The phenomenon of narcissism has been described for around 2000 years when the myth of Narcissus was documented in ancient Greece. In this myth, the beautiful young hunter Narcissus rejected the love of the nymph Echo, which is why he was deemed by the gods to fall in love with his own mirror image. His self-centeredness ultimately led Narcissus to the fate of a tragic death. Thus, on a metaphorical level, this early myth already reflects two aspects of narcissism that are currently being extensively studied, namely grandiose and vulnerable narcissism. The ancient concept of narcissism was picked up and refined by psychodynamic theorists, who regarded narcissism as both a self-regulatory mechanism and a personality disposition (Freud, 1914; Kernberg, 1975; Kohut, 1977), and was first included as a personality disorder in the third edition of the Diagnostic and Statistical Manual of Mental Disorders (American Psychiatric Association, 1980). At the same time, the concept of narcissism was also adapted for personality research within general, nonclinical personality variation (Raskin & Hall, 1979).

It was not until the 2000s, however, that research on narcissism attracted a broader scientific and public interest, which is presumably due to cultural changes in the new millennium. Popular writings such as Jean Twenge’s (2006) “Generation Me” or Twenge and Campbell’s (2009) “Narcissism Epidemic” render a rather pessimistic picture of western societies becoming increasingly self-focused and entitled, while being less bound to social and moral norms. Large-scale studies of trait changes initiated a lively scientific debate on whether narcissism scores increase in western cultures (Barry & Lee-Rowland, 2015; Donnellan, Trzesniewski, & Robins, 2009; Twenge, Konrath, Foster, Campbell, & Bushman, 2008; Wetzel et al., 2017) and are dependent on cultural orientation (Jauk, Breyer, Kanske, & Wakabayashi, 2021; Vater et al., 2018). The strong interest in the past decades also fueled a thorough research program on what exactly constitutes narcissism as a personality trait and as a clinical disorder, what are its antecedents, and its personal and interpersonal consequences. To this end, only a few personality constructs have received attention from so many different perspectives as is the case for narcissism. Different but overlapping theories have been put forward from social/personality psychology, clinical psychology, and psychiatry (Cain et al., 2008).

**Figure 1.**
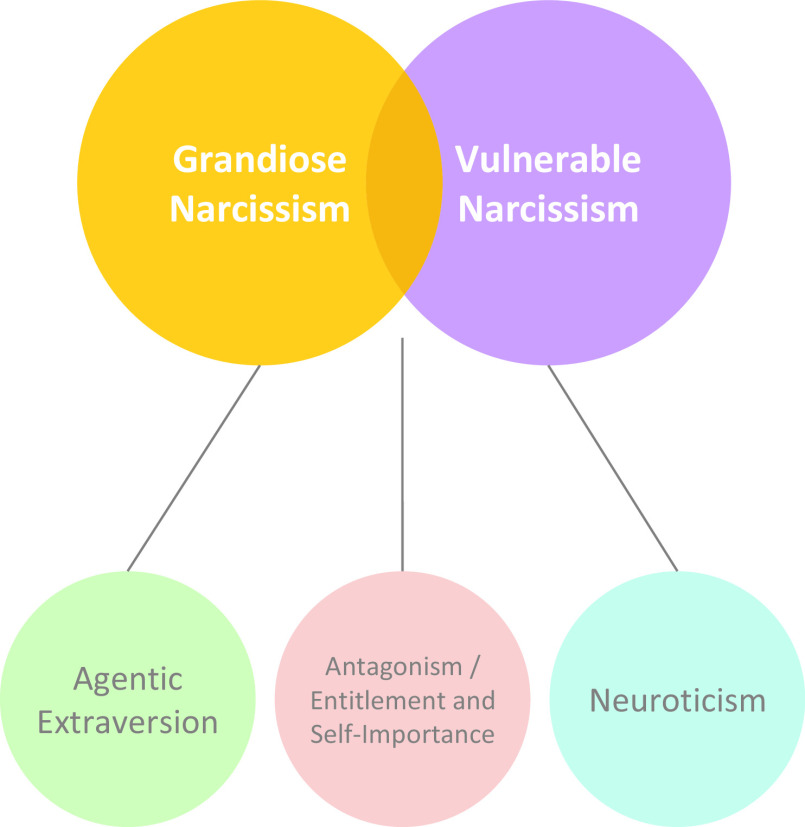
Structural model of narcissism, adapted and synthesized from the Trifurcated Model (Miller et al., 2016; Weiss et al., 2019) and the Narcissism Spectrum Model (Krizan & Herlache, 2018).

There is now an emerging consensus that narcissism is multifaceted in nature and different expressions of the phenomenon can be discerned (see Figure 1). While all forms of narcissism are characterized by pronounced feelings of self-importance and entitlement, grandiose and vulnerable narcissism have been recognized as separable yet related expressions of narcissism (Krizan & Herlache, 2018; Miller et al., 2016; Weiss et al., 2019). These manifest in distinct experiential and behavioral patterns of either self-assuredness and dominance, or self-consciousness and withdrawal. Despite their seemingly opposing manifestations, both share overtly or covertly expressed attitudes of being special and entitled to special privileges (Krizan & Herlache, 2018; Miller et al., 2016; Pincus & Lukowitsky, 2010). The overarching mechanism of narcissistic functioning is the maintenance of an inflated self by means of characteristic intra- and interpersonal self-regulatory strategies (Back et al., 2013; Morf & Rhodewalt, 2001).

Also, in recent years, neuroscience has begun to unveil aspects of narcissism that are not commonly apparent in self-report research. Neuroscience methods offer a particularly promising view into narcissistic personality functioning as they might be less influenced by cognitive biases and response styles that can render pure self-report studies problematic. These include, among others, overestimation of emotion-related and social competencies in narcissism (Ames & Kammrath, 2004; Jauk, Freudenthaler, et al., 2016; John & Robins, 1994; Lobbestael et al., 2016; Mota et al., 2019; Zajenkowski et al., 2018), which are directly relevant to the studies reviewed here.

The goal of this review is to summarize the current neuroscience investigations on narcissism and integrate them with conceptual models from personality and clinical psychology to highlight the possible contributions of neuroscience to the understanding of narcissism. In the first part, we will provide an overview of the different conceptions of narcissism and their commonalities, differences, and manifestations in different aspects of experience and behavior. We will then, in the second part, review neuroscience investigations of the different conceptions of narcissism, and highlight their potential to gain a deeper understanding of the respective constructs. Finally, in the third and fourth parts, we provide a conceptual integration and a model of the neural bases of self-related and interpersonal processes in narcissism, and close with recommendations for future research in the personality neuroscience of narcissism.

We structure our review in *intra*personal and *inter*personal aspects of narcissism, as these are conceived as the two major self-regulatory pathways in research on narcissism (Back et al., 2013; Morf & Rhodewalt, 2001), and more generally, two major dimensions of personality functioning in prevailing models of personality pathology (Alternative Model for Personality Disorders [AMPD] in the DSM-5; see Table 1; Bender, Morey, & Skodol, 2011; Personality Disorders Model in the ICD-11, Tyrer, Mulder, Kim, & Crawford, 2019). Intrapersonal functioning, following the DSM-5 AMPD, concerns aspects of *identity* – a stable and coherent sense of self, stable self-esteem, and capacity to experience, tolerate, and regulate affect – and *self-regulation* – goal pursuit, utilization of internal standards of behavior, and self-reflection. Interpersonal functioning concerns aspects of *empathy* ^1^ – understanding others’ experiences and motivations, tolerance of differing perspectives, understanding of social causality – and *intimacy* – connection with others, desire and capacity for closeness, and cooperative behavior (American Psychiatric Association, 2013; Bender et al., 2011).

**Table 1.** Dimensions of intra- and interpersonal functioning

Intrapersonal functioning		Interpersonal functioning	
Identity	Self-regulation	Empathy/social cognition	Intimacy
– Stable and coherent self	– Goal pursuit	– Understanding others‘ experiences and motivations	– Connection with others
– Stable self-esteem	– Utilization of internal standards of behavior	– Tolerance of differing perspectives	– Desire and capacity for closeness
– Capacity to experience, regulate, tolerate affect	– Self-reflection	– Understanding of social causality	– Cooperative behavior

Personality functioning model is adapted from the DSM-5 Alternative Model of Personality Disorders (AMPD; American Psychiatric Association, 2013; Bender et al., 2011). The term “social cognition” was added by us based on prevailing neuroscience models (see section 2.1).

### 1.1 Grandiose narcissism

When hearing the term “narcissism”, most people think of exaggerated self-worth, feelings of superiority, admiration seeking, entitlement, and arrogance (Buss & Chiodo, 1991). These common associations actually very closely match the definition of grandiose narcissism as a personality trait, which encompasses self-importance and entitlement – the antagonistic core of narcissism – alongside extraverted and socially dominant behavior (Krizan & Herlache, 2018; Miller et al., 2016).

The concept of narcissism as a trait was originally devised from the diagnostic criteria for Narcissistic Personality Disorder (NPD) in the third edition of the Diagnostic and Statistical Manual of Mental Disorders (American Psychiatric Association, 1980), and adapted for the assessment of narcissism in the general, nonclinical population in the *Narcissistic Personality Inventory* (NPI; Raskin & Hall, 1979, 1981). The original criteria included (1) a grandiose sense of self-importance, (2) fantasies of unlimited success, power, etc., (3) exhibitionism, (4) responding to criticism with either indifference or rage, (5) entitlement, (6) exploitativeness, (7) relationships vacillating between idealization and devaluation, and (8) lack of empathy. Importantly, these criteria focus strongly on narcissistic grandiosity, and only implicitly tap into narcissistic vulnerability (see section 1.2). Items of the NPI encompass statements such as “I think I am a special person”, “I am more capable than other people”, or “I have a natural talent for influencing people” (Raskin & Terry, 1988). The NPI has become the most widely used measure of grandiose narcissism, the total score displays good reliability and validity with respect to expert ratings (Miller et al., 2014), and the vast majority of the neuroscience studies on grandiose narcissism reviewed here used the NPI as an indicator.

#### 1.1.1 Intrapersonal characteristics

Individuals high in grandiose narcissism report high self-esteem (Campbell, 2001) and score high on different indicators of mental health (Sedikides et al., 2004) as well as life satisfaction (Egan et al., 2014). They display marked self-enhancement in various domains, which means that they rate themselves as more intelligent or attractive, for instance, than it might be expected on the basis of objective tests or others’ ratings (Campbell et al., 2002; Gabriel et al., 1994; Grijalva & Zhang, 2016; Grosz et al., 2017). Self-enhancement is also evident regarding social and emotional capacities in grandiose narcissism (Ames & Kammrath, 2004; Jauk, Freudenthaler, et al., 2016; John & Robins, 1994; Lobbestael et al., 2016; Mota et al., 2019; Zajenkowski et al., 2018), which indicates that individuals high in grandiose narcissism hold pronounced positive illusions about their intra- and interpersonal emotional abilities. In line with their superior and independent self-construal, individuals high in grandiose narcissism report discomfort only in light of achievement failure, but not in the light of social rejection (Besser & Priel, 2010). Taken together, self-descriptions of individuals high in grandiose narcissism render a picture of highly self-assured individuals, which is most likely due to the agentic-extraverted aspects of narcissism (Kaufman et al., 2020).

However, behavioral and experimental studies also unveil fragilities in the seemingly overly positive grandiose-narcissistic personality. These encompass, for instance, feelings of self-alienation or a weak sense of self (Kaufman et al., 2020), proneness to addictive behaviors (Jauk & Dieterich, 2019), and heightened variability in mood and self-esteem (Geukes et al., 2017; Rhodewalt et al., 1998). Particularly, experiencing negative interpersonal events can lead to sudden declines in self-esteem (Zuckerman & O’Loughlin, 2009). Variability in self-esteem is more related to antagonistic (i.e., disagreeable) than agentic (i.e., extraverted, socially dominant) aspects of narcissism (Geukes et al., 2017).

#### 1.1.2 Interpersonal characteristics

Individuals high in grandiose narcissism are extraverted, socially bold (Holtzman, Vazire, & Mehl, 2010), and also charming (e.g., Back, Schmukle, & Egloff, 2010). Accordingly, they can be highly successful in social contexts, which is, for instance, evident in the domain of interpersonal attraction (Dufner et al., 2013; Jauk, Neubauer, et al., 2016). This picture conforms to the “happy face” of grandiose narcissism (Rose, 2002), which is most likely due to its agentic-extraverted aspects (Kaufman et al., 2020). However, the short-term social success of individuals high in grandiose narcissism is also likely to be accompanied by long-term interpersonal problems, such as, for instance, in romantic relationships (Wurst et al., 2017). Interpersonal problems associated with grandiose narcissism are thought to arise from heightened self-focus and reduced empathy (Morf & Rhodewalt, 2001), both of which are perceived as core pillars of the grandiose narcissism construct and are more related to its antagonistic aspects (Kaufman et al., 2020). Though a number of studies addressed the relationship between grandiose narcissism and empathy (e.g., Giammarco & Vernon, 2014; Wai & Tiliopoulos, 2012), the exact nature of lowered empathy in narcissism is still a matter of debate (cf. Baskin-Sommers et al., 2014). Recent behavioral research points into the direction of a reduced propensity to share others’ emotional states, rather than a reduced ability to do so (Hepper et al., 2014).

### 1.2 Vulnerable narcissism

Besides grandiose narcissism, increasing attention has been paid to vulnerable narcissism in the past decades (e.g., Fossati et al., 2009). While vulnerable aspects of narcissism have long been hypothesized to be overt or covert parts of the broader phenomenon of narcissism (Pincus & Lukowitsky, 2010), the social/personality study of narcissism delineated vulnerable narcissism as an independent trait – the “second face” of narcissism (Wink, 1991). Vulnerable narcissism is uncorrelated to grandiose narcissism at a trait level in the general population, but might blend into grandiosity at high levels of grandiose narcissism (Jauk & Kaufman, 2018; Jauk, Weigle, et al., 2017). To date, only two neuroscience studies explicitly addressed vulnerable narcissism in that they used independent measures to assess this construct. Nevertheless, the construct may also be relevant to the interpretation of studies on grandiose and pathological narcissism as well as NPD.

Individuals high in vulnerable narcissism typically present very differently from those high in grandiose narcissism: vulnerable narcissistic individuals are anxious, defensive, and avoidant (Hart et al., 2017; Miller et al., 2012). Yet, despite the outward presentation of self-consciousness, those high in vulnerable narcissism also share the common antagonistic core of entitlement and self-importance (Krizan & Herlache, 2018; Miller et al., 2016). A commonly used measure of vulnerable narcissism, the Hypersensitive Narcissism Scale (HSNS; Hendin & Cheek, 1997), encompasses items such as “I easily become wrapped up in my own interests and forget the existence of others” or “I am secretely [sic!] ‘put out’ when other people come to me with their troubles, asking me for my time and sympathy“ (p. 592).

#### 1.2.1 Intrapersonal characteristics

While grandiose narcissism is closely tied to extraversion, vulnerable narcissism is closely related to neuroticism and – depending on the scale used – introversion (Jauk, Weigle, et al., 2017; Kaufman et al., 2020; Miller et al., 2016). Consequently, vulnerable narcissism is linked to experiencing less positive and more negative affect (Miller et al., 2011, 2018), and individuals high in vulnerable narcissism display a variety of internalizing symptoms such as anxiety and depression (Euler et al., 2018; Kaufman et al., 2020; Miller et al., 2011, 2018). They report lower self-esteem (Brookes, 2015; Miller et al., 2018; Rose, 2002) and lower life satisfaction (Rose, 2002). Taken together, psychological and behavioral correlates of vulnerable narcissism clearly point to the “unhappy” face of narcissism (Rose, 2002).

#### 1.2.2 Interpersonal characteristics

Contrary to those high in grandiose narcissism, those high in vulnerable narcissism are more sensitive to social rejection than achievement failure (Besser & Priel, 2010). Paradoxically, while individuals high in vulnerable narcissism tend to put great weight on being accepted by others, they also display lowered empathy (Lannin et al., 2014) and compassion (Luchner et al., 2011) for others. Vulnerable narcissism is further related to lower self-report perspective-taking (Honeycutt et al., 2014) and emotion understanding performance (Vonk et al., 2015). Taken together, the pattern of interpersonal dynamics in vulnerable narcissism points to the desire of being accepted by others, accompanied by the fear of being rejected, which is in line with higher attachment anxiety in vulnerable narcissism (Rohmann et al., 2012). Excessive self-focus and impaired interpersonal functioning likely amplify this fear in terms of negative social outcomes.

### 1.3 Pathological narcissism

Pathological narcissism, as an operationally defined and quantifiable construct, was introduced by Pincus and colleagues in recognition of the problem that previously existing self-report measures of (grandiose) narcissism mostly focused on adaptive qualities, and maladaptive as well as vulnerable aspects might have been underrepresented (Pincus et al., 2009). Consequently, pathological narcissism is defined as comprising both grandiose and vulnerable aspects (Pincus et al., 2009; Pincus & Lukowitsky, 2010). While these seemingly opposing experiential and behavioral tendencies may appear hard to reconcile at the first glance, Pincus and Lukowitsky (2010) assert that grandiose and vulnerable self-states can fluctuate or co-occur in highly narcissistic individuals (Wright & Edershile, 2018), which is illustrated vividly in clinical case descriptions (Pincus et al., 2014). The current standard measure of pathological narcissism is the *Pathological Narcissism Inventory* (PNI; Pincus et al., 2009). It encompasses facets related more to narcissistic grandiosity, namely *entitlement rage* (anger when entitled expectations are not met), *exploitativeness* (manipulative behavior in pursuit of own goals), *grandiose fantasy* (imagining grandiose self), and *self-sacrificing self-enhancement* (self-enhancement by means of seemingly altruistic behavior), as well as facets related more to narcissistic vulnerability, which are *contingent self-esteem* (dependence upon others’ regard), *hiding the self* (not showing own needs and weaknesses), and *devaluing* (devaluing others who do not meet their own needs). The grandiose and vulnerable factors are intrinsically related in the PNI (Pincus et al., 2009). This makes it conceptually and empirically different from scales designed exclusively for the assessment of either grandiose or vulnerable narcissism, which are unrelated in the general population (Jauk, Weigle, et al., 2017).

#### 1.3.1 Intrapersonal characteristics

Individuals high in pathological narcissism report, among others, lower self-esteem, higher aggression, and higher shame (Morf et al., 2017; Pincus et al., 2009), higher psychological distress, depressive symptoms, and lower life satisfaction (Morf et al., 2017), as well as higher self-harming behavior (Dawood et al., 2018). Behavioral studies of pathological narcissism found more positive intrapersonal and future-oriented thoughts, but more negative thoughts in general (Kanske, Sharifi, Smallwood, Dziobek, & Singer, 2017). This reflects two important aspects of pathological narcissism, namely grandiose fantasy on the one hand, and on the other hand, vulnerable or devaluing aspects (Pincus et al., 2009).

#### 1.3.2 Interpersonal characteristics

Pathological narcissism is associated with fearful or preoccupied attachment (both characterized by a negative model of the self; Fossati, Feeney, Pincus, Borroni, & Maffei, 2015; Morf et al., 2017) and an array of self-report interpersonal problems ranging from cold dominant to overly nurturant behavior (Pincus et al., 2009). In daily interactions, individuals high in pathological narcissism are reactive to both status threat and rejection sensitivity (Roche et al., 2013). Pathological narcissism goes along with serious treatment problems in clinical samples, including suicidal ideation and behavior (Pincus et al., 2009). Pathological narcissism is negatively related to dispositional empathy (Pincus et al., 2009), which can be explained along the line that both of its constituent features, grandiosity, and vulnerability, are accompanied by lowered empathy (see sections 1.1.2 and 1.2.2). Perspective-taking is lowered in pathological narcissism in social decision-making (Böckler et al., 2017), but not in a self-report measure (Morf et al., 2017). This indicates that pathological narcissism goes along with similar self-serving bias as grandiose narcissism when it comes to interpersonal abilities (see section 1.1.1). Taken together, the experiential and behavioral findings related to pathological narcissism point to a pattern of psychological maladjustment characterized by opposing tendencies of grandiosity and vulnerability, significant personal distress, and maladaptive interpersonal behavior.

### 1.4 Narcissistic personality disorder

The diagnostic category of NPD emerged in the DSM-III mainly from psychodynamic theory and case studies (Ronningstam, 2009). Since its first inclusion, the diagnostic criteria (mentioned above) have been revised. In the latest edition of the Diagnostic and Statistical Manual of Mental Disorders (DSM-5; American Psychiatric Association, 2013), the categorical diagnosis of NPD is defined by at least five out of the following nine criteria: (1) grandiose sense of self-importance, (2) fantasies of unlimited success, power, etc., (3) thinks he/she is special and unique, (4) requires excessive admiration, (5) entitlement, (6) exploitativeness, (7) lack of empathy, (8) envious/thinks others are envious, and (9) arrogance. Contrary to the developments in the research field, the definition of NPD in the DSM-5 is strongly focused on grandiosity (Pincus & Lukowitsky, 2010; Ronningstam, 2009) and might – in its present form – best be understood as an extreme manifestation of grandiose narcissism, which is supported by concurrent validity evidence (Miller et al., 2014). This does not mean that vulnerable aspect cannot accompany grandiosity (which is also explicitly acknowledged in the DSM), but they do not constitute a necessary condition for an NPD diagnosis. For the purpose of the present review, it is most important to note that the terms pathological narcissism and NPD, despite their similar connotation, cannot be used interchangeably. While pathological narcissism, sensu Pincus and Lukowitsky (2010), refers to concurrent grandiosity *and* vulnerability, NPD is a clinical diagnosis for individuals with extreme levels of grandiosity, which may or may not be accompanied by vulnerability.

The prevalence of NPD is generally considered low, as compared to other mental disorders. Prevalence estimates in the general population range between 0 and 1% (Roepke & Vater, 2014) or 0–5% (Ronningstam, 2009), the DSM-5 provides an estimated range between 0 and 6.2% in community samples (American Psychiatric Association, 2013). Systematic empirical research on NPD is generally very sparse, putatively because patients are hard to find, and hard to recruit. We summarize some of the available evidence in the following.

#### 1.4.1 Intrapersonal characteristics

One of the most prevailing notions on NPD is the view that outward grandiosity is a façade to hide an underlying, fragile self (Akhtar, 1989), also known as the “mask” model of narcissism. One hypothesis that can be derived from such a mask model view posits that high explicit self-esteem should be accompanied by low implicit self-esteem in narcissism (cf. Kuchynka & Bosson, 2018). Empirical studies, however, did not find evidence for lower implicit self-esteem among NPD patients (Marissen et al., 2016; Vater et al., 2013). On the contrary, one of the studies unexpectedly found lower explicit self-esteem of NPD patients as compared to healthy controls (Vater et al., 2013). It has to be noted, though, that NPD patients do not commonly seek treatment until they experience a major breakdown, which might explain their lowered explicit self-esteem (Vater et al., 2013).

#### 1.4.2 Interpersonal characteristics

Reduced empathy is one of the diagnostic criteria of NPD. Consequently, NPD patients show less mirroring of emotions, and less emotional contagion than healthy controls (Ritter et al., 2011). The picture is more diverse when it comes to measures of perspective-taking, also referred to as Theory of Mind (Frith & Frith, 2005). One study found that NPD patients report lower perspective-taking, but do not differ from healthy controls on performance measures (Ritter et al., 2011). However, a different study suggests that NPD patients perform worse than healthy controls on an emotion recognition task, and that they are unaware of this deficit (Marissen et al., 2012). Recently, a study on NPD patients reported lowered interview-assessed mindreading performance as compared to healthy controls, but similar performance to patients with other personality disorders (Bilotta et al., 2018). A review highlights the role of motivation-based disengagement (propensity) versus deficit-based (capacity) lack of interpersonal functions in NPD (Baskin-Sommers et al., 2014). It is argued that not all forms of reduced interpersonal functioning are created equal, and can either be a consequence of a marked self-focus despite generally intact social–affective and cognitive functions (reduced propensity), or also a lack of fundamental interpersonal skills (reduced capacity; ibid.).

The reduced interpersonal functioning of NPD patients can have significant consequences not only for people in their surroundings, but also for themselves. NPD patients with severe levels of pathology are known as being hard to treat (Kernberg, 2007), and indeed, psychotherapists experience feeling unappreciated or devalued by these patients and report disengagement from the therapeutic process (Tanzilli et al., 2015).

### 1.5 Summary: grandiosity and vulnerability in narcissism

According to current models, narcissism entails both grandiose and vulnerable aspects, which manifest in experiential and behavioral patterns of self-assuredness and dominance on the one hand, and insecurity and reactivity on the other (see Figure 1; Krizan & Herlache, 2018; Weiss et al., 2019). In the general population, self-reports of trait grandiosity and vulnerability are unrelated or only weakly positively related, which points to relatively independent personality configurations – sharing a common core of self-importance and entitlement/antagonism (Krizan & Herlache, 2018; Weiss et al., 2019) – at a global level (Jauk & Kaufman, 2018; Jauk, Weigle, et al., 2017). However, it has been posited that those high in grandiosity can fluctuate between grandiose and vulnerable states (e.g., Wright & Edershile, 2018), which is evident in informant reports (Gore & Widiger, 2016), increases in correlations among trait measures of grandiosity and vulnerability with increasing grandiosity (Jauk & Kaufman, 2018; Jauk, Weigle, et al., 2017), and ecological momentary assessment of state grandiosity and vulnerability (Edershile & Wright, 2020). Grandiosity and vulnerability are further intrinsically related in measures assessing primarily maladaptive, pathological aspects of narcissism (such as the PNI; Pincus et al., 2009), and vulnerable self-states can be observed in NPD (American Psychiatric Association, 2013; Ronningstam, 2009), a personality disorder characterized by extreme grandiosity (Miller et al., 2014). Taken together, while grandiose and vulnerable narcissism can be described in terms of dissociable personality configurations at a trait level, there is also increasing evidence that these two “faces” of narcissism go hand in hand – particularly at high levels of grandiosity. An interesting question for the neuroscience of intrapersonal functioning in narcissism might thus be to identify conditions under which individuals high in grandiose narcissism display reactions which are indicative of vulnerability, even if not directly evident in self-reports. Another question concerns interpersonal functioning, which is altered in all expressions in narcissism discussed above. We elaborate on these questions in the following section 2.1.

## 2. Neuroscience of Narcissism

### 2.1 Current research questions for the neuroscience of narcissism

The overarching goal of this review is to highlight the possible contributions of neuroscience to the understanding of narcissism in its different expressions. Though self-report and behavioral research on narcissism have acquired a great deal of knowledge in the past decades, some questions still remain open. Broadly, these concern two fundamental aspects of personality functioning: (1) intrapersonal, self-related characteristics of narcissism and (2) interpersonal, other related characteristics of narcissism.

(1) It has long been hypothesized that narcissism, particularly grandiose narcissism and NPD as its clinical expression, entail vulnerable aspects, which need not necessarily be overtly expressed (e.g., Pincus & Lukowitsky, 2010). The mask model of narcissism posits that grandiosity is a façade to mask an underlying fragile self (Akhtar, 1989); that is, that grandiosity compensates for underlying vulnerability. Whether or not grandiosity is indeed causally related to underlying vulnerability, as this model implies, clinicians working with narcissistic patients are inclined to agree with their coexistence (in correlational terms). In their seminal review, Pincus and Lukowitksy (2010) assert that “many contemporary clinical experts on narcissistic personality disorder now recognize that grandiose self-states oscillate or co-occur with vulnerable self-states and affective dysregulation” (p. 428). Similarly, in her review on NPD, Ronningstam (2009) states that: “the narcissistic individual may fluctuate between assertive grandiosity and vulnerability” (p. 113). Following this perspective, one might assume that individuals high in grandiose narcissism should hold implicit negative self-views along their explicit positive self-views (cf. Kuchynka & Bosson, 2018). However, this view is not commonly supported by systematic research. Self-report studies on grandiose narcissism find positive correlations with explicit (Campbell et al., 2002) and also implicit self-esteem (Campbell et al., 2007), and research on NPD suggests no difference in implicit self-esteem between NPD patients and controls (Marissen et al., 2016; Vater et al., 2013).

The question remains, thus, if and how negative self-views are represented in individuals high in grandiose narcissism, and neuroscience might help to shed light on the respective mechanisms. In the long run, this might also contribute to understanding state changes between grandiosity and vulnerability discussed above (e.g., Edershile & Wright, 2020). Neuroscience studies targeting intrapersonal functions can investigate the neurophysiological reactions to situations in which the individual is exposed to ego threat, for instance, in terms of achievement failure, social exclusion, or confrontation with self-referential stimuli. These might induce stronger involuntary stress responses in individuals with higher levels of narcissism (cf. Coleman et al., 2019). Indicators that are known to be sensitive to these responses include, among others, autonomic measures such as skin conductance (Jacobs et al., 1994) or blood pressure (Ulrich-Lai & Herman, 2009) as well as neuroendocrine markers such as cortisol level (Kirschbaum et al., 1993). Using neuroimaging methods, activity within regions of the salience network (comprising the anterior insula [AI] and dorsal anterior cingulate cortex [dACC]; Bressler & Menon, 2010) has been associated with the processing of aversive stimuli that induce conflict, pain, or negative affect (de la Vega et al., 2016; Shackman et al., 2011; Somerville et al., 2006).^2^ These indicators might thus unveil involuntary vulnerable reactions and are less prone to cognitive bias than pure self-report measures. Moreover, habitual experiential and behavioral tendencies related to narcissism might manifest in chronic functional alterations in the mentioned systems, which can be revealed by studies of baseline function.

(2) Another controversial aspect of narcissism concerns the exact nature of altered interpersonal functioning observed in different expressions of narcissism. Interpersonal functioning includes – alongside more complex constructs such as attachment (see Table 1) – fundamental social–affective and social–cognitive functions frequently termed *empathy* and *perspective-taking*. Empathy refers to sharing others’ affective and emotional states, whereas perspective-taking (or Theory of Mind) circumscribes the capacity to cognitively represent others’ mental states (de Vignemont & Singer, 2006; Frith & Frith, 2005). While both are sometimes subsumed under empathy as an umbrella term (being also referred to as affective and cognitive empathy; e.g., Baron-Cohen & Wheelwright, 2004), they can be selectively impaired and draw on distinct neural networks (Stietz et al., 2019; see also below). Importantly, empathy and perspective-taking display considerable state variation and can depend on contextual and also motivational factors (ibid.), raising the question of whether altered interpersonal functioning in narcissism is more a question of reduced capacity (lower ability) or propensity (lower engagement; cf. Baskin-Sommers et al., 2014).

Narcissistic individuals are known to display lowered empathy (indeed, limited empathy is among the defining criteria of grandiose narcissism; see section 1.1), and there is also evidence for impaired social cognition in NPD (Marissen et al., 2012). However, behavioral evidence suggests that limited empathy in narcissism might be more due to lower propensity than capacity (Hepper et al., 2014), and similar mechanisms are being discussed for putative perspective-taking deficits in NPD (Baskin-Sommers et al., 2014). It might thus be the case that altered interpersonal functioning in narcissistic individuals is more related to the consequences of a self-focused and antagonistic interpersonal style (Krizan & Herlache, 2018; Miller et al., 2016) than ability deficits per se. Again, neuroscience might help to gain a closer understanding of the mechanisms involved in interpersonal functioning in narcissism. A neuroscience approach seems particularly fruitful given that global self-reports of empathy and perspective-taking (as measured by the frequently used Interpersonal Reactivity Index, for instance; Davis, 1983) display only low-to-moderate correlation with task-based ratings and corresponding neural activation (Hildebrandt et al., 2021), which points to differences between global self-perceptions of typical behavior and interpersonal capacities assessed under laboratory conditions. This gap might be even stronger for individuals high in narcissism, whose self-ratings of emotional competencies, in general, deviate substantially from task-based assessments (Jauk, Freudenthaler, et al., 2016; Mota et al., 2019).

Neuroscience research on interpersonal functioning has delineated empathy from perspective-taking; the two main capacities that enable access to others’ inner, mental states, thus, providing crucial ground for successful social interaction. Empathic reactions to others’ emotional states are commonly observed in the same regions that are activated during the firsthand experiences of these states (Kanske, 2018). Thus, for instance, empathy for another’s aversive experience, be it pain, disgust or unfair treatment, activates the AI and the (middle) anterior cingulate cortex (parts of the salience network; see above), which are also activated during firsthand aversive stimulation (Corradi-Dell’Acqua et al., 2016; Oosterwijk et al., 2017). This supports the view that empathy for others’ emotional states might be regarded as an internalization of these states (Kanske, 2018), or in other words, we literally feel what others are feeling when empathizing. With respect to narcissism, this might make alexithymia, in terms of the general incapacity to experience emotions, a prime candidate as a mediating factor (Valdespino et al., 2017).

Perspective-taking, or Theory of Mind, draws on a different neuronal network than empathy (Kanske, Böckler, Trautwein, & Singer, 2015). This network comprises medial prefrontal regions, the temporoparietal junction, the precuneus/posterior cingulate cortex, as well as the temporal poles and superior temporal sulcus (Bzdok et al., 2012; Molenberghs et al., 2016; Schurz et al., 2021); structures that partially overlap with the default mode network (Mars et al., 2012). Activation within the network implicated in perspective-taking directly corresponds to task performance (Kanske et al., 2015). Most importantly for the present review, neurophysiological research showed that empathy and perspective-taking are clearly independent and distinguishable processes, both on a behavioral and neurophysiological basis (Kanske, Böckler, Trautwein, Parianen Lesemann, & Singer, 2016). Intra- and interindividual variations in the two capacities are independent as well (Stietz et al., 2019). This means that empathic functioning and perspective-taking could be selectively altered in different expressions of narcissism. Neuroscience research might unveil alterations in the involved networks during empathy and perspective-taking tasks, during rest, and in structural measures.

### 2.2 Literature search and inclusion strategy

For this review, we considered quantitative empirical journal articles on grandiose and vulnerable narcissism, pathological narcissism, and NPD using neuroimaging, neurophysiological, or neuroendocrine measures. For the inclusion, we required a standardized assessment of narcissism that targets one of the constructs introduced above. We searched the academic databases Scopus and PubMed, complemented by Google Scholar searches, for the following terms in the article abstracts: narciss* + neuro*, bio*, physio*, EEG, *MRI, and *imaging. After the exclusion of 3 empirical articles using qualitative methods and 4 studies that did not use neuroscience methods, we obtained a set of 35 empirical journal articles. Of those journal articles, one (Yang, Sedikides, Gu, Luo, Wang, Yang, et al., 2018) targeted the relatively novel construct of communal narcissism (an expression of agentic, narcissistic strivings through communal means, such as being the most helpful person; Gebauer, Sedikides, Verplanken, & Maio, 2012). As this study is to date the only neuroscience investigation of the communal narcissism construct, and the construct itself is still relatively new,^3^ we include this study in the discussion section, rather than the main part of the review. Another study (Kelsey et al., 2002) used subscales of a multidimensional personality scale, the Bell Object Relations and Reality Testing Inventory (BORRTI; Bell, 1995) for the assessment of overt and covert narcissism (which were sometimes equated with grandiose and vulnerable narcissism). As it was not possible to evaluate the convergent validity of the used subscales (egocentricity and alienation) with more widely used measures of grandiose and vulnerable narcissism, and also the authors themselves discuss the validity of the measure critically (Kelsey et al., 2002), we excluded this study from our review. We excluded one further study as the indicator of narcissism was too indirect to allow for comparisons with the rest of the literature (peer ratings of grandiosity were transformed into measures of sociometric preference in the crucial analysis involving a neuroendocrine marker; Bukowski, Schwartzman, Santo, Bagwell, & Adams, 2009). Table 2 summarizes the remaining 32 studies. In addition to these published studies, we also found two unpublished doctoral dissertations meeting the abovementioned criteria (Krusemark, 2009; Noser, 2017), which we also included in the main part of this review and Table 2, leading to a total of 34 studies. Finally, our literature search identified one hypothesis proposal (Jankowiak-Siuda & Zajkowski, 2013), two review articles that each cover parts of the research reviewed here (Coleman et al., 2019; Di Sarno et al., 2018), one conceptual paper (George & Short, 2018), and four book chapters (Konrath & Bonadonna, 2014; Elizabeth A. Krusemark, 2012, 2018; Schulze & Roepke, 2014), which do not present empirical data but rather integrate previous studies. We include these works in section 3 of the present review.

**Table 2.** Summary of neuroscience studies on narcissism

Study	Narcissism construct (measure)	Sample	Study type	Measure	Design	Intra-/interpersonal	Results
Cascio et al. (2015)	Grandiose (NPI)	Nonclinical, 43 male adolescents	Experiment	fMRI	Social exclusion (Cyberball)	Intra	Higher activation in dACC, sgACC, and AI following social exclusion in more narcissistic young men
Cheng et al. (2013)	Grandiose (NPI)	Nonclinical, 67 female students	Baseline	Cortisol, alpha-amylase	Three-day longitudinal	-	Higher cortisol and alpha-amylase related more strongly to negative affect among highly narcissistic women
Chester & DeWall (2016)	Grandiose (NPI-16)	Nonclinical, 30 students (15F/15M)	Experiment	fMRI	Social Exclusion (Cyberball)	Intra	High narcissism and high dACC activation interactively related to aggression (administering noise blasts)
Chester et al. (2016)	Grandiose (NPI-16)	Nonclinical, 50 students (32F/18M)	Structure	DTI	Correlational	-	Narcissism negatively related to white matter integrity (fractional anisotropy) in the frontostriatal pathway
Dane et al. (2018)	Grandiose (NPI)	Nonclinical, 25 male students	Experiment	Cortisol, testosterone	Lying on camera	Intra	High narcissism associated with decreases in cortisol (nonsignificant) and increases in testosterone
Edelstein et al. (2010)	Grandiose (NPI)	Nonclinical, 90 students (46F/44M)	Experiment	Cortisol	Trier Social Stress Test	Intra	Highly narcissistic men showed stronger cortisol responses in the TSST
Fan et al. (2011)	Likely Pathological (NI)	Nonclinical, 11 high (6F/5M) versus 11 low (7F/4M) NI scorers	Experiment	fMRI	Empathize with emotional faces	Inter	High narcissism associated with activation differences in right AI, dorsolateral prefrontal cortex, premotor cortex, and posterior cingulate cortex. In the right AI, lower positive signal change from the control to the empathy condition for high narcissism
Feng et al. (2018)	Grandiose (NPI)	Nonclinical, 168 students (59F/109M)	Baseline	rs-fMRI	Correlational	-	Narcissism associated with functional connectivity differences in amygdala, dorsal and ventral mPFC, dACC, and ventrolateral prefrontal cortex
Grapsas et al. (2020)	Grandiose (CNS)	Nonclinical, 123 children (68F/55M)	Experiment	EMG	Bogus social hierarchy feedback	Intra	High narcissism associated with stronger increases in corrugator supercilii activity (negative affect) during status loss and stronger increases in zygomaticus major activity (positive affect) as well as corrugator supercilii activity during status gain
Jauk, Benedek, et al. (2017)	Grandiose (NPI)	Nonclinical, 21 high (8F/13M) versus 22 low (14F/8M) NPI scorers	Experiment	fMRI	Self-face-viewing	Intra	Highly narcissistic men show higher activation in dACC and vACC for own face
Kelsey et al. (2001)	Grandiose (NPI)	Nonclinical, 20 high versus 20 low male NPI scorers	Experiment	ECG, skin conductance	Active and passive coping with loud tones	Intra	Highly narcissistic men show greater skin conductance habituation and stronger heart rate deceleration
Krusemark (2009)	Grandiose (NPI)	Nonclinical, 20 high versus 20 low NPI scorers	Experiment	EEG	Self-serving versus non-self-serving attributions after false performance feedback	Intra	Highly narcissistic participants make more self-serving attributions after receiving positive performance feedback, accompanied by weaker event-related activity in reaction to stimuli, but stronger activity in reaction to feedback
Lee et al. (2020)	NPD	Clinical, 98 psychiatric outpatients, thereof 15 meeting NPD criteria (3F/12M) versus 68 community controls (35F/33M) and 29 (19F/10M) psychiatric outpatients without personality disorders	Baseline	8-hydroxy-2'-deoxyguanosine (8-OH-DG)	Correlational	-	8-OH-DG, an indicator of oxidative stress burden, is higher in NPD and borderline patients than patients with other PDs and clinical as well as nonclinical controls
Lobbestael et al. (2014)	Grandiose (NPI) and vulnerable (HSNS)	Nonclinical, 100 male students	Experiment	Testosterone	Adapted Taylor Aggression Paradigm	Inter	Grandiose but not vulnerable narcissism related to aggression (administering noise blasts) and increases in testosterone
Mao et al. (2016)	Pathological (PNI)	Nonclinical, 176 (91F/85M)	Structure	Surface-based morphometry	Correlational	-	Narcissism associated with lower thickness and volume in the right dorsolateral prefrontal cortex; lower thickness right inferior frontal cortex; lower volume in right postcentral gyrus and the left mPFC
Marcoux et al. (2014)	NPD	Clinical, 11 male NPD outpatients versus 13 male community controls	Experiment	EEG	Observation of others’ pain	Inter	Increased somatosensory resonance with others’ pain as indicated by EEG P3 gating of tactile stimulation
Mead et al. (2018)	Grandiose (NPI)	Nonclinical, 192 young adults (92F/199M)	Experiment	Testosterone (dependent measure: NPI)	Social power manipulation	Inter	Individuals with higher baseline testosterone show higher NPI entitlement/exploitativeness scores when assigned social power
Mück et al. (2020)	Grandiose (NARQ)	Nonclinical, 59 students (42 F/17M)	Experiment	EEG	Self-face viewing	Intra	Agentic and antagonistic aspects of grandiose narcissism modulate early (P1) and later (N170) EEG responses
Nenadic et al. (2015)	NPD	Clinical, 6 male NPD patients versus 48 male controls	Structure	VBM, DTI	Correlational	-	NPD patients show lower gray matter volume in the right middle frontal gyrus, left mPFC/ACC, left middle occipital cortex, left fusiform/inferior temporal cortex, right superior temporal cortex, and left lingual gyrus. Lower fractional anisotropy in right frontal lobe, right anterior thalamic radiation, right anterior temporal lobe, left anterior/lateral temporal lobe, and right brain stem
Noser (2017)	Grandiose (DTDD)	Nonclinical, 109 adult men	Baseline	Testosterone	Correlational	-	Narcissism positively associated with baseline testosterone
Pfattheicher (2016)	Grandiose (SDT)	Nonclinical, 128 male students	Baseline	Cortisol, Testosterone	Correlational	-	Narcissism positively associated with baseline cortisol and testosterone
Reinhard et al. (2012)	Grandiose (NPI)	Nonclinical, 106 students (79F/27M)	Baseline	Cortisol	Correlational	-	Narcissism is associated with baseline cortisol in men, but not women (also in women when only considering entitlement/exploitativeness NPI factor)
Scalabrini et al. (2017)	Grandiose (PNI)	Nonclinical, 32 young men	Experiment	fMRI	Social touch	Inter	Grandiosity negatively related to AI activation during human versus mannequin touch anticipation
			Baseline	rs-fMRI	Correlational	-	Grandiosity positively related to long-range temporal correlation in AI
Schulze et al. (2013)	NPD	Clinical, 17 NPD patients (16 out-, 1 inpatient; 12M/5F) versus 17 community controls (12M/5F)	Structure	VBM	Correlational	-	NPD patients have lower gray matter volume in left AI, rostral and median cingulate cortex, and dorsolateral prefrontal cortex as well as mPFC
Sommer et al. (2009)	Grandiose (NPI)	Nonclinical, 74 students (42F/32M)	Experiment	Blood pressure, heart rate	Rejection imagery	Intra	Individuals with higher NPI entitlement/exploitativeness scores have higher systolic blood pressure during rejection imagery and rejection story completion
Stenstrom et al. (2018)	Pathological (PNI)	Nonclinical, 104 young men	Experiment	Testosterone	Money exposure	Intra	Money exposure leads to testosterone increases only for those with low PNI scores, but not high PNI scores
Sylvers et al. (2008)	Grandiose (SCID II and SCATI)	Nonclinical, 93 students (sex-balanced)	Experiment	skin-conductance reactivity, pre-ejection period, respiratory sinus arrhythmia	countdown-to-aversive-stimuli, affective pictures	Intra	Narcissism not associated with skin conductance reactivity or pre-ejection period during countdown-to-aversive-stimuli. Narcissism negatively related to respiratory sinus arrhythmia and pre-ejection period shortening during happy pictures
Wang et al. (2006)	NPD	Clinical, 17 NPD outpatients (8F/9M) versus 26 controls from different sources (18F/8M)	Baseline	Auditory evoked potentials	Correlational	-	No differences in auditory evoked potentials for NPD
Wardecker et al. (2018)	Grandiose (NPI)	Nonclinical, 366 students (48.2% F)	Baseline	Cortisol	Correlational	-	No association between NPI overall or factor scores and cortisol levels; no interaction with sex
Woodman et al. (2011)	Grandiose (NPI)	Nonclinical, 42 students (21F/21M)	Experiment	Heart rate, ergometer cycling performance	Ergometer cycling under low or high identifiability	Intra	Individuals higher on narcissism show higher performance and heart rate under high identifiability
Yang et al. (2015)	Grandiose (NPI)	Nonclinical, 326 students (176F/150M)	Baseline	rs-fMRI	Correlational	-	Increased anticorrelation between default mode structures and dorsal attention network structures in more narcissistic women, decreased anticorrelation in men
			Structure	VBM	Correlational	-	Positive association between narcissism and regional gray matter volume in superior parietal lobe in women, but not in men
Yang et al. (2018)	Grandiose (NPI)	Nonclinical, 49 young adults (11F/38M)	Experiment	EEG	Monetary gambling task	Intra	Higher narcissism associated with riskier decisions and weaker P3 response following outcomes of high-risk decisions
Zhang et al. (2016)	NPD	Clinical, 14 NPD outpatients (4F/10M) versus 25 controls from different sources (12F/13M)	Experiment	EEG	Facial emotion processing	Inter	No differences for facial emotion processing in EEG components between NPD patients and controls, but negative modulation of depression on P2 to neural and happy faces in NPD
Zhang et al. (2015)	Grandiose (NPI) and vulnerable (HSNS)	Nonclinical, 227 students (188F/39M)	Experiment	Respiratory sinus arrhythmia	Mock job interview	Intra	Respiratory sinus arrhythmia was not related to grandiose or vulnerable narcissism, but moderated the association of vulnerable narcissism and self-report emotion regulation

The studies are described in section 2 of the main text. F, female; M, male. Abbreviations of narcissism measures: CNS, Childhood Narcissism Scale; DTDD, Dark Triad Dirty dozen; HSNS, Hypersensitive Narcissism Scale; NARQ, Narcissistic Admiration and Rivalry Questionnaire; NI, Narcissism Inventory; NPD, narcissistic personality disorder; NPI, Narcissistic Personality Inventory; NPI-16, abbreviated 16-item – version; PNI, Pathological Narcissism Inventory; SCATI, Short Coolidge Axis II inventory; SCID II, structured clinical interview for DSM-IV, personality disorders; SDT, short dark triad. Abbreviations of neuroscience measures: ECG, Electrocardiogram; EEG, Electroencephalogram; EEG, Electromyogram; DTI, diffusion tensor imaging; fMRI, functional magnetic resonance imaging; rs-fMRI, resting-state fMRI; VBM, voxel-based morphometry. Abbreviations of brain structures: ACC, anterior cingulate cortex; dACC, dorsal ACC; vACC, ventral ACC; sgACC, subgenual ACC; AI, anterior insula; mPFC, medial prefrontal cortex.

As Table 2 shows, among the 34 studies, 21 studies used experimental paradigms, in which participants were confronted with stimuli or tasks. Ten studies used baseline measures of brain activity or neuroendocrine markers, and five studies used structural brain imaging measures.^4^ We classified the experimental paradigm studies as either primarily *intra*personal (self-related paradigms) or *inter*personal (other related paradigms) in focus. This classification is in line with social/personality models of narcissism (e.g., Morf & Rhodewalt, 2001) and with prevailing models of personality functioning, such as the DSM-5 AMPD (American Psychiatric Association, 2013). Though this distinction may not appear straightforward in each case (such as, for instance, the touch anticipation paradigm used by Scalabrini et al., 2017), we capitalized on whether the main focus of the paradigm is to manipulate intrapersonal, self-regulatory (e.g., emotion regulation), or interpersonal (e.g., empathizing, emotion recognition) psychological processes.

As Table 2 further shows, the vast majority of neuroscience research investigated the construct of grandiose narcissism (25 studies). Three studies investigated pathological narcissism.^5^ Only two studies, to date, explicitly focused on vulnerable narcissism in which separate measures were used to assess vulnerability (though some discuss narcissistic vulnerability as a potential mechanism of the observed results). All studies on grandiose or pathological narcissism used nonclinical samples, mostly comprised of students. Six studies investigated NPD in patients.

In the following, we will summarize the findings of these studies grouped in functional, baseline, and structural studies of the different narcissism constructs. Table 2 provides an overview of all studies including sample characteristics. We will then integrate this research into a general discussion, in which we also consider prior reviews and book chapters on the topic.

### 2.3 Experimental studies

#### 2.3.1 Studies targeting intrapersonal functions

Almost all among the 15 studies of intrapersonal functions in narcissism reviewed here investigated the construct of grandiose narcissism in nonclinical samples; one study also assessed vulnerable narcissism, and one study focused on pathological narcissism. Of those studying grandiose narcissism, all but three used the NPI (see section 1.1) as an indicator of grandiosity. One of the central questions in neuroscience studies targeting intrapersonal functions in narcissism is if and to what extent individuals high in narcissism are sensitive to ego threat in that they display stronger stress responses facing potentially threatening self-relevant information (see section 2.1). The majority of studies investigating intrapersonal, self-related characteristics of grandiose narcissism demonstrate that individuals high in grandiose narcissism are indeed sensitive to situations involving actual or anticipated ego threat, as discussed in the following.

Sommer and colleagues (2009) studied the consequences of interpersonal rejection and found that individuals higher in the entitlement/exploitativeness facet of the NPI displayed stronger stress reactions in terms of increases in systolic blood pressure when listening to rejection stories and completing rejection stories, as opposed to acceptance stories. At a subjective level, when rejection by a romantic partner had to be imagined (as opposed to rejection by a friend), participants scoring high on the NPI reported greater anger. Similarly, Cascio and colleagues (2015) had male adolescents perform a Cyberball game during functional magnetic resonance imaging (fMRI). Participants believed they were interacting with real others, while actually the game was programmed to first include, and then exclude participants. Stronger activation for exclusion was found in a network referred to by the authors as the “social pain network” (p. 335) – largely overlapping with the salience network – comprising the dACC, the AI, and the subgenual anterior cingulate cortex in more narcissistic young men. Interestingly, while previous self-report research showed that individuals high in grandiose narcissism are sensitive to achievement failure but not social exclusion (Besser & Priel, 2010), and narcissism was not related to self-reports of social exclusion in this study, grandiose narcissism correlated with a brain activity pattern previously shown to relate to feelings of social exclusion (Eisenberger, 2003). Activation in the dACC was also related to social exclusion on a meta-analytic basis (Rotge et al., 2015), though a recent meta-analysis observed dACC activation only in a minority of studies (Mwilambwe-Tshilobo & Spreng, 2021), and the amount of dACC involvement might depend on the used task (Mwilambwe-Tshilobo & Spreng, 2021; Rotge et al., 2015). The activation patterns observed in this study thus led the authors to conclude that “narcissists’ social pain [is] seen only in the brain” (Cascio et al., 2015, p. 335). The neuroimaging results obtained for social exclusion in this study are further similar to those observed for social exclusion in people with low self-esteem (Onoda et al., 2010), and individuals with borderline personality disorder (Wrege et al., 2019), which may suggest similar neural mechanisms for narcissistic individuals and those with low self-esteem or severe deficits in personality functioning. The social exclusion line of research was continued in a later study using the Cyberball paradigm (Chester & DeWall, 2016). Focusing again on the dACC as part of the salience network, referred to as the brain’s “alarm system” (p. 362) by the authors, it was found that dACC activation was higher during social exclusion, and that grandiose narcissism and dACC activation interactively predicted the willingness to administer loud noise blasts to the bogus Cyberball partners. While narcissism did not show a significant main effect on dACC activation in this study, there was a significant interaction between narcissism and anxious attachment on dACC activation. The authors conclude that grandiose narcissism, paired with neural markers of rejection distress, leads to aggressive behavior.

Another study demonstrated the increased sensitivity of individuals high in grandiose narcissism to ego threat using a neuroendocrine marker: Edelstein, Yim, and Quas (2010) had participants either undergo the Trier Social Stress Test (TSST; Kirschbaum, Pirke, & Hellhammer, 1993) or a control condition. The TSST reliably induces strong stress responses by requesting participants to perform a public speech and a mental arithmetic task in front of an audience. Still, individual differences in the extent of these stress responses can be related to psychological variables. Stress responses were quantified by means of saliva cortisol levels at several time points. Highly narcissistic men showed higher peaks and prolonged stress responses as compared to low narcissistic men in the TSST. No difference was found in women. Similar, albeit weaker results were obtained for ratings of negative affect. A similar study investigated the effects of a mock job interview, as a social stressor, on respiratory sinus arrhythmia (RSA) in young adults (Zhang, Wang, You, Lü, & Luo, 2015). Participants’ task was to introduce themselves to a committee of strangers, and were told that they would be videotaped and evaluated afterward. Pre-, peri-, and post-interview measures of RSA were assessed as an indicator of emotion regulation capacity. Of note, this study is to date one of the two, which used independent indicators of grandiose and vulnerable narcissism (NPI and HSNS). RSA was not per se related to either narcissism measure, but vulnerable narcissism moderated the associations between RSA reactivity and a self-report measure of emotion regulation in the way that those higher in vulnerable narcissism who also displayed lower RSA decreases showed stronger emotion regulation difficulties. The authors interpret these effects in terms of RSA as a potentially protective factor in vulnerable narcissism.

Finally, using a related experimental approach, yet a different physiological measure, Grapsas and colleagues (2020) recently investigated the effects of social stress in the form of status threat. A large sample of children (mean age of 10 years) performed a social hierarchy task on a simulated social media platform, being either randomly assigned a low or a high social status in the bogus ranking. Children with higher levels of grandiose narcissism (assessed via the Childhood Narcissism Scale, a measure drawing strongly on grandiose narcissism; Thomaes et al., 2008) reacted with stronger increases in negative affect (measured via electromyography of the corrugator supercilii, involved in frowning) during status loss, and stronger increases in positive affect (measured via electromyography of zygomaticus major, involved in smiling) and negative affect during status gain. The results show that sensitivity to social status in relation to narcissism can already be observed at a young age.

So far, it can be concluded that situations involving social rejection or exclusion, threat of evaluation (TSST), or status threat induce stronger stress responses (increased blood pressure, saliency network activation, cortisol release) or aggressive behavior (administering noise blasts) in individuals high in grandiose narcissism. One study, however, did not find such an effect using RSA (Zhang et al., 2015). Of note, grandiose narcissism amplifies negative affective responses in face of ego threat even in children (Grapsas et al., 2020). While this research points to increased stress responses for individuals higher in narcissism in situations that are supposed to be threatening for most of us, further research shows that even stimuli that are not intrinsically threatening induce similar responses: Jauk, Benedek, and colleagues (2017) had extreme groups of individuals either high or low in grandiose narcissism perform a visual self-viewing paradigm during fMRI. Viewing own faces, as compared to viewing friends’ or strangers’ faces, was accompanied by higher activation in the dACC and also the ventral ACC (vACC) in highly narcissistic men – findings that are similar to those described above for social exclusion (Cascio et al., 2015). The authors concluded that self-referential stimulation induces conflict (expectancy violation) or negative affect in grandiose-narcissistic men. Though this interpretation seems to be generally in line with the understanding of the dACC as a region implicated in adaptive control in the light of potential threat (de la Vega, Chang, Banich, Wager, & Yarkoni, 2016; Kanske & Kotz, 2011; Shackman et al., 2011; Somerville, Heatherton, & Kelley, 2006), it needs to be noted that neither the dACC nor the salience network, in general, does have a single, unitary function (Seeley, 2019). The findings can thus only be interpreted in the particular context, which – considered together with the above-reviewed studies on social exclusion (Cascio et al., 2015; Chester & DeWall, 2016) – suggests that individuals high in narcissism show stronger saliency-related responses in the light of self-referential material, putatively more indicative of negative than positive anticipatory processes (de la Vega et al., 2016; Shackman et al., 2011). A recent EEG study by Mück and colleagues (2020) used a similar self-face viewing paradigm: participants either viewed pictures of their own face, a celebrity, or a stranger. EEG differences were investigated in two different components: the P1 component, which reflects an early stage of face processing and is higher when valence is ascribed to the stimulus, and the N170, which reflects a later stage of face processing and is commonly higher for the own face as compared to others’ faces.^6^ The moderating effect of agentic and antagonistic aspects of grandiose narcissism (admiration and rivalry; see Back et al., 2013) was investigated. Individuals high on agentic narcissism or low on antagonistic narcissism displayed a lower P1 response (for self vs. celebrity), and individuals higher on antagonistic narcissism displayed a lower N170 response (for self vs. stranger). The authors put forward different interpretations for these findings, the first of which is that agentic narcissism is associated with an inhibition of early attention toward the own face in order to maintain a grandiose self-image, whereas antagonistic narcissism is associated with increased attention at later stages for similar self-protection goals. While the different psychological and neuroscience measures used in this study and the previously discussed study by Jauk, Benedek, and colleagues (2017) make it hard to reconcile both studies’ results, it can be tentatively hypothesized that the effects observed for antagonistic narcissism, indicative of increased later stage processing facing potential ego threat, might be related to the increased activity in parts of the saliency network observed earlier. In any case, the results of both studies show that narcissism modulates neural responses to self-related material (own face), presumably in terms of increased sensitivity or vigilance for ego threat, even though this material is not intrinsically threatening.

This interpretational bias might even be stronger when it comes to pathological narcissism: Stenstrom and colleagues (2018) investigated the effects of money exposure on testosterone level in young men varying on pathological narcissism. Participants’ task was to sort either banknotes (experimental condition, which was hypothesized to increase testosterone levels) or paper (control condition; between-Ss). Only men scoring low on pathological narcissism showed the expected increases in testosterone after sorting banknotes, but those high on pathological narcissism did not. The authors interpret this as a result of the intimidating effect the money exposure might have on men high on pathological narcissism, who perform a downward comparison threatening their ego. Finally, the effect of anticipated ego threat is apparent in a study by Woodman and colleagues (2011), who had participants perform a physical performance task, cycling on an ergometer, either under conditions of low or high social identifiability. Students were cycling in groups of three; in the low-identifiability condition, they were told that only the group performance would be made public at the university, whereas in the high-identifiability condition, they were told that individual performance would also be published. Cycling performance and heart rate increased under the high-identifiability condition for those high in grandiose narcissism. While this is not directly an indicator of experienced stress (as increases in heart rate might be explained by increases in performance), it indicates that individuals higher in narcissism are willing to invest more in the light of social evaluation, which is in line with general approach orientation in relation to grandiose narcissism (Spencer et al., 2018).

However, stimuli that are self-related but *not self-relevant* do not generally elicit stronger reactions in relation to narcissism: the first physiological study of grandiose narcissism investigated autonomic responses during active and passive coping with stressful stimuli (Kelsey et al., 2001). Extreme groups of men low versus high in grandiose narcissism were exposed to aversive auditory stimuli (loud tones), which could either be avoided (active coping condition) or were unavoidable (passive coping condition). Men high in grandiose narcissism displayed – at the same time – lower anxiety (greater skin conductance habituation) and greater vigilance (stronger heart rate deceleration) in response to aversive stimuli, which was consistent with their lower self-reported state anxiety. Interestingly, for these men, pre-ejection period shortening did not differ between active and passive coping, which the authors interpreted as fight/flight response independent of actual coping demands. Results indicate that men high in grandiose narcissism display cool but vigilant reactions to stressful stimuli, which bear similarities to the psychopathy construct (Kelsey et al., 2001). This line of research was extended by Sylvers and colleagues (2008), who used a countdown-to-aversive-stimuli (white noise blast) task to study physiological reactions related to grandiose narcissism. Grandiose narcissism was assessed using both structured interviews and self-reports based on the current^7^ DSM criteria. In contrast to the authors’ expectations and the findings reported by Kelsey and colleagues (2001), narcissism was not associated with any physiological changes, which might indicate lowered distress (skin conductance hyporeactivity or pre-ejection period shortening). Instead, antisocial traits were associated with these indicators. The authors attribute the discrepant results to differences in the assessment of narcissism, and critically discuss the use of clinical assessments in nonclinical samples. For the purpose of the present review, however, it seems most important to note that none of the studies observed stronger stress reactions in relation to narcissism.^8^ Stimuli that are self-related but not self-*relevant*, such as loud tones or noise blasts, do not lead to stress reactions (Kelsey et al., 2001; Sylvers et al., 2008), or even elicit emotionally cool, prepared reactions (Kelsey et al., 2001), which points to some overlap between narcissism and the psychopathy construct, traditionally characterized by fearlessness (Lykken, 1995; see also Hare & Neumann, 2008).

This is further evident in two studies targeting deceptive and risky behavior: Dane, Jonason, and McCaffrey (2018) recently investigated the neuroendocrine reactions of men differing in grandiose narcissism (NPI) to the effects of lying. Participants were instructed to behave like effective liars, and were videotaped while telling two true and one lie statement about themselves.^9^ The pre-post – comparison of salivary cortisol and testosterone revealed that (1) men higher in narcissism showed a pattern of higher baseline cortisol (which is in line with the finding of Reinhard, Konrath, Lopez, & Cameron, 2012 as well as Pfattheicher, 2016, discussed in section 2.4) and post-lie decreases in cortisol, though this effect was not significant in the small sample. These results thus differ from those reported by Edelstein, Yam, and Quas (2010), who observed increases following the TSST, and point to the moderating influence of the subjective stress level, or also level of enjoyability induced by the task. While the TSST was experienced as stressful by more narcissistic male participants (Edelstein et al., 2010), the lying task was actually perceived as enjoyable by more narcissistic participants (Dane et al., 2018). An opposite pattern of results was observed for testosterone in this study, with more narcissistic men displaying increases in testosterone, and less narcissistic men displaying decreases, which points to enjoyment and the self-aggrandizing function of deceiving others. One recent EEG study investigated risk-taking processes in relation to grandiose narcissism (Yang et al., 2018). Participants performed a monetary gambling task, where they could choose between low- and high-risk options. At the behavioral level, individuals higher in narcissism made more high-risk choices in trials that were preceded by other high-risk trials. At the neurophysiological level, no differences in feedback-related negativity emerged between more and less narcissistic individuals, which means that error monitoring did not differ between individuals with lower and higher narcissism. However, highly narcissistic individuals showed a weaker P3 response following outcomes of high-risk decisions, which the authors interpret as evidence for reduced action updating in narcissism leading to further risk-taking behavior as a consequence of reduced feedback learning. Taken together, these studies again point to some overlap with the psychopathy construct, which is also characterized by a bold interpersonal style (including lying; Hare & Neumann, 2008) and the reduced ability to learn from previous experiences (Galang, 2010).

Finally, an unpublished EEG study investigating the neural correlates of self-enhancement (Krusemark, 2009) found that individuals high in grandiose narcissism make more self-serving attributions after false performance feedback in a working memory task (i.e., attributing positive feedback internally, irrespective of actual task performance), and that this is accompanied by relatively weaker event-related activity in widespread regions of the cortex. Activation related to the feedback itself, on the contrary, was accompanied by stronger neural responses. The author interprets this pattern of results as a more shallow processing in relation to biased attributions, but a greater responsiveness to evaluative information. Considered together with the studies reviewed above, this may be seen as supporting the picture of grandiose-narcissistic individuals displaying overly confident, hyporeactive responses when it comes to decision-making (Yang, Sedikides, Gu, Luo, Wang, & Cai, 2018) and attribution of success (Krusemark, 2009), alongside increased responsiveness to potentially self-relevant information (Grapsas et al., 2020; Jauk, Benedek, et al., 2017; Krusemark, 2009).

#### 2.3.2 Studies targeting interpersonal functions

Six studies investigated the neuroscience correlates of interpersonal functions in narcissism. Contrary to the studies reviewed above, which mostly focused on grandiose narcissism, these works span a more diverse palette of different expressions of narcissism including also pathological narcissism and NPD, and use heterogeneous experimental paradigms. We review these studies beginning with more high-level emotion processing paradigms (e.g., empathize with emotional faces), turning then to more low-level paradigms (e.g., sensory processing of emotional stimuli), and finally to social–psychological accounts (e.g., interactions among experimental variations, personality, and neuroendocrine markers).

Fan and colleagues (2011) conducted the first fMRI study on interpersonal functions in narcissism, focusing on the well-documented empathic alterations. The study used extreme groups of low and high narcissistic individuals according to the Narcissism Inventory (NI; Denecke & Hilgenstock, 1989), which likely assesses pathological narcissism.^10^ Participants either viewed emotional faces or, as a control condition, smoothed faces, and were instructed to empathize with these faces. Highly narcissistic individuals showed activation differences in the right AI, dorsolateral prefrontal cortex, premotor cortex, and in the posterior cingulate cortex; regions overlapping with those identified by a meta-analysis for empathy; particularly in the AI (Lamm et al., 2011). In the right AI, highly narcissistic individuals showed a lower positive signal change from the control to the empathy condition (Fan et al., 2011). Importantly, the highly narcissistic group also showed higher alexithymia scores, which lead the authors to conclude that “the apparent difficulty in accessing the own emotions, as suggested by our finding of increased alexithymia, may lead to problems in simulating the other’s affective states as our neural findings may be interpreted” (p. 1649). This may point to a general difficulty of experiencing emotional states as a basis of altered empathic functioning in narcissism, as expressed in a recent model (Valdespino et al., 2017), and as is the case with increased alexithymia in other conditions as well (Bird et al., 2010; Hoffmann et al., 2016). A further fMRI study investigated the effects of social touch, which may be considered another high-level interpersonal paradigm, in narcissism: Scalabrini and colleagues (2017) had a sample of male participants either touch a human hand or a mannequin hand in the MRI scanner. Human touch anticipation was accompanied by lower activation in the right AI in individuals scoring high on grandiose narcissism (grandiose factor of the PNI^11^). Interestingly, narcissistic grandiosity not only correlated with task-related, but also with resting-state activity. Narcissistic grandiosity mediated the association between spontaneous, resting-state, and task-related activity in the AI. The authors thus concluded that “narcissism could function as a factor mediating between internal processing, related to the self, and external sensory information related to the social world” and “may be related to an increased internal predisposition accompanied by a motivation-based disengagement from social processing” (p. 10). Taken together, both studies show alterations of activity in the AI, a key node of empathic processing within the salience network (Lamm et al., 2011).

Turning now to more low-level emotion processing paradigms, Marcoux and colleagues (2014) used a somatosensory gating paradigm to investigate emotional perceptiveness in NPD. In this paradigm, a steady-state stimulation (repetitive tactile stimulation on the hand) is modulated by viewing either images depicting a painful situation of another person, which should evoke empathic reactions, or non-painful images as a control condition. Somatosensory gating is operationalized by the reduction of somatosensory activity related to the tactile stimulation, indexed by the EEG P3 amplitude, in painful versus non-painful images; i.e., individuals who are supposed to display weaker empathic reactions should show weaker gating effects. Contrary to what could be expected, the male NPD patients showed an increased somatosensory gating effect compared to controls, and this effect was not attributable to different subjective pain thresholds. The authors interpreted their findings in the way that narcissistic men were “feeling but not caring” (p. 341); i.e., they displayed stronger somatosensory resonance, but did not report higher empathic concern or distress, and lower perspective-taking, in a trait self-report measure. These results may be seen as supporting the notion of reduced propensity, not capacity, of empathic alterations in NPD (Baskin-Sommers et al., 2014) in a low-level sensory processing paradigm. In other words, it can be tentatively concluded that low-level other directed processing capability is not generally affected in NPD, but is probably attenuated for motivational reasons. In a similar study, Zhang and colleagues (2016) investigated EEG event-related potentials during facial emotion processing in different personality disorder groups. The sample of NPD patients – who displayed higher anxiety and, partly, higher depression than controls in this study – were found to take longer time to respond to happy faces (but not other emotions), which were intermixed with landscape pictures. At a neurophysiological level, though no main effects for narcissism were found across different electrodes, components, and measures (latency, amplitude), P2 amplitude to neutral and happy faces was negatively related to depression in NPD patients. The authors interpreted the effects in the way that depression amplifies self-focus in narcissism. This self-focus reduces other directed processing particularly in the light of neutral or positive emotion, which is noncongruent to the own depressed state. This interpretation is consistent with the notion of state-dependent alterations – in this case, depression level – of interpersonal functioning in narcissism (Baskin-Sommers et al., 2014) for a low-level paradigm. Thus, taken together with the previous study by Marcoux and colleagues (2014), it can be concluded that low-level processing of other related stimuli is not generally impaired in NPD (Marcoux et al., 2014; Zhang et al., 2016), but can be impaired in states of amplified self-focus, such as in depression (Zhang et al., 2016), particularly when emotions are positive or noncongruent to the own emotion (Zhang et al., 2016; for similar findings from depression research, see Hoffmann et al., 2016).

Turning now to the social–psychological line of research on narcissism, Lobbestael and colleagues (2014) investigated behavioral aggression and testosterone in grandiose and vulnerable narcissism: a sample of young men performed an adapted Taylor Aggression Paradigm, in which participants were made believe that they were playing a competitive game against a human opponent. After each round, noise blasts of varying intensities and durations could be administered to the bogus opponent. Results showed that young men higher in grandiose, but not vulnerable narcissism, displayed more aggression in administering noise blasts, and along with that also increases in testosterone level. The authors interpret these findings in terms of a higher propensity of grandiose-narcissistic individuals to act aggressively toward others, which also reflects in characteristic neuroendocrine changes. Finally, one recent study also took a social–psychological approach to study interpersonal functioning in narcissism in relation to testosterone: Mead and colleagues (2018) assigned participants varying on grandiose narcissism (NPI) randomly to either a power condition, where they were made believe they would exert control over others in the following group task, or a control condition, where they were just informed that they were going to take part in the group task. Those participants in the power condition, who additionally had high baseline testosterone, displayed higher scores on the entitlement/exploitativeness factor of the NPI, and reported higher willingness to misuse power. Thus, contrary to other studies reviewed here, this study speaks to the antecedents of grandiose narcissism in terms of individual biological disposition (testosterone level) and social influences (power manipulation). It thus shows that grandiose narcissism cannot only be regarded as a trait that exerts its influence on other variables, but also as a state that can itself be influenced by biological and social factors. This finding is in line – though opposite in causal direction – with the results of Dane and colleagues (2018) as well as Lobbestael and colleagues (2014), who reported increases in testosterone levels in more narcissistic men after lying to others/behaving aggressively toward others. Taken together, these studies point to dynamic interactions between narcissism, testosterone, and situations involving the exertion of social power, deception, or aggression.

### 2.4 Baseline function studies

We identified 10 studies that investigated different biophysiological measures of baseline functioning in narcissism: six studies used measures of neuroendocrine markers including cortisol, other neuroendocrine markers targeting habitual stress levels, or testosterone, three investigated resting-state fMRI (one of them is the already discussed social touch study by Scalabrini et al., 2017), and one study used auditory evoked potentials in EEG.^12^ Except for two studies, which were carried out on NPD patients, all other studies investigated continuous differences in grandiose narcissism in relatively large samples.

Studies targeting neuroendocrine markers of habitual stress mostly speak to elevated stress in relation to narcissism, partially moderated by other variables: Reinhard and colleagues (2012) carried out the first study on the association between a standardized measure of grandiose narcissism (NPI) and baseline cortisol levels. In a relatively large sample of young individuals, they observed an interaction among sex and narcissism in the way that narcissistic men have higher baseline cortisol across two time points separated by a 25-minute period. The effect sizes were substantial with correlations around *r* = .40. Critically, this effect could not be explained by potentially confounding psychological variables (positive/negative mood, stress, social support, or relationship status). The effects were stronger for the entitlement/exploitativeness factor than for other factors of the NPI, and were also evident in females when considering only this factor. This shows that particularly those aspects of grandiose narcissism that are most maladaptive (Ackerman et al., 2011) are associated with chronic stress. Subsequently, two studies attempted to indirectly or directly replicate the results observed by Reinhard and colleagues (2012): the first study successfully replicated the associations between grandiose narcissism and baseline measures of cortisol, and extended it by measures of testosterone among young men (Pfattheicher, 2016). It was found that higher levels of narcissism – but not of its near neighbor’s psychopathy or Machiavellianism – were related to higher levels of both baseline cortisol and baseline testosterone as assessed by two salivary probes separated by 6 min. The author interprets the findings in the way that men with higher levels of narcissism are at the same time responsive to social threat (cortisol) and socially dominant (testosterone). The second study, however, did not replicate the findings: Wardecker and colleagues (2018) investigated the association of grandiose narcissism (NPI) and baseline cortisol in a large sample of young women and men. They did neither observe any overall associations between narcissism and cortisol, nor interactions with sex or effects for specific NPI factors, as reported in the original study. Though the replication attempt was well powered, methodological differences such as the saliva sampling procedure could still have contributed to the failure to replicate. Finally, an unpublished study replicated the associations between grandiose narcissism (assessed using the Dark Triad Dirty Dozen, which focuses on grandiosity, but also correlates with vulnerability; Jonason & Webster, 2010; Küfner et al., 2015) and baseline testosterone: Noser (2017) observed a correlation (*r* = 0.22) of narcissism and salivary testosterone in a large sample of adult men, but not of its neighboring personality dimensions psychopathy or Machiavellianism.

Two more studies investigated cortisol and further neuroendocrine markers in narcissism: Cheng and colleagues (2013) studied the relationships between cortisol, alpha-amylase, and grandiose narcissism in young women who provided saliva samples and ratings of positive and negative affect for three consecutive days. Both cortisol and alpha-amylase were higher in those who were high in grandiose narcissism and experienced more negative affect. The authors conclude that narcissism acts as a moderator in the relationship between perceived stress and negative health outcomes, as evident in cortisol (related to activity of the hypothalamic-pituitary-adrenal axis) and alpha-amylase (related to sympathetic nervous system activity). Finally, a recent study (Lee et al., 2020) investigated baseline levels of 8-hydroxy-2'-deoxyguanosine (8-OH-DG), an indicator of oxidative stress at a cellular level (which has been associated with a variety of mental disorders; e.g., Salim, 2014), in patients with different personality disorders, other mental disorders, and controls. Results showed higher levels of 8-OH-DG in patients with NPD and borderline personality disorder; no such effects were observed for other personality disorders. The effects were independent of relevant covariates including age, gender, alcohol, and cigarette use, major depression, and posttraumatic stress disorder. The authors interpret the findings in terms of increased biological stress responses to interpersonal hypersensitivity. Considered together with the studies by Reinhard and colleagues (2012), Pfattcheicher (2016), Cheng and colleagues (2013), as well as the TSST study by Edelstein and colleagues (2010), these results provide relatively consistent evidence for exaggerated biological stress responses (cortisol, alpha-amylase, and 8-OH-DG) in relation to grandiose narcissism or NPD as its extreme expression, though one study did not find such associations (Wardecker et al., 2018). The exact nature of stress responses might depend on sex or gender (see section 4), the expression of entitlement/exploitativeness, which most closely reflects the antagonistic core of the construct (Krizan & Herlache, 2018; Weiss et al., 2019) and is considered most maladaptive (Ackerman et al., 2011), and subjective stress levels as moderating factors.

Three studies investigated resting-state functional brain connectivity by means of fMRI. The first of these (Yang et al., 2015) studied functional connectivity differences in relation to grandiose narcissism (NPI) in a large sample of young women and men. The authors identified brain structural correlates of narcissism in the first step (see section 2.5), and then used regions in the right superior parietal lobe and the frontal eye fields – parts of the dorsal attention network – as seeds for subsequent functional connectivity analyses. In women, increased anticorrelations between the dorsal attention network and the default mode network were found as a function of narcissism. In men, the opposite pattern emerged, with decreased anticorrelation between the dorsal attention network and the default mode network as a function of narcissism. The authors interpret the effects in terms of a proneness toward outward-directed admiration seeking in more narcissistic women and loose boundaries between internal and external events in more narcissistic men. The second and most recent resting-state fMRI study of grandiose narcissism (NPI, Chinese Likert scale adaption) attempted to predict trait narcissism in a large sample of young individuals by means of machine learning applied to functional connectivity maps (Feng et al., 2018). A network specific for subjects high in grandiose narcissism consisting of key nodes in the amygdala, the lateral and medial prefrontal cortex (mPFC), and the anterior cingulate cortex could be identified. Validation analyses showed that connectivity information within this network predicts grandiose narcissism scores in an independent sample of participants. While the network identified in this study is complex, interestingly, it encompasses some of the regions that were also found to discriminate between lower and higher narcissistic individuals in experimental functional imaging studies, above all the dACC (Cascio et al., 2015; Chester & DeWall, 2016; Jauk, Benedek, et al., 2017) as part of the salience network. Individuals high in grandiose narcissism further display altered connectivity within regions commonly implicated in emotion processing (given the role of the amygdala, as part of the limbic system, in fear conditioning; LeDoux, 2000) and regions belonging to the default mode network (mPFC), for which structural changes can be observed (see section 2.5). Finally, in the already discussed combined resting-state and task-based fMRI study by Scalabrini and colleagues (2017), a power law exponent analysis, measuring long-range temporal correlations in resting-state data, was conducted in ROIs that were functionally identified in the social touch experiment (human hand vs. mannequin hand: left postcentral gyrus, right culmen, and right AI; the latter only in interaction with grandiose narcissism). A significant positive association between grandiose narcissism and long-range temporal correlation was found in the AI, which the authors interpret in terms of increased self-focus in more grandiose individuals, particularly concerning aspects of the bodily and interoceptive self. Taken together, these studies show that grandiose narcissism can be related to intrinsic functional connectivity differences in the resting brain, and that the involved regions – parts of the default mode and salience networks – overlap with those implicated in imaging studies of intra- and interpersonal functioning (see section 3.3 for integrative discussion).

Finally, one EEG study used auditory evoked potentials to investigate cerebral information processing differences in several personality disorder groups, among them a group of NPD patients (Wang et al., 2006). The neural responses to auditory stimuli of different amplitude were studied. It was hypothesized that certain personality disorder groups would display an “augmenting” evoked potential pattern indicative of sensation seeking and low serotonin levels. Such a pattern was found for patients with histrionic personality disorder, but no differences emerged for NPD. As with the studies reviewed in sections 2.3.1 and 2.3.2 above, it seems that low-level sensory processing is not generally altered in narcissism.

### 2.5 Structural imaging studies

Four structural imaging studies on narcissism were carried out so far, investigating gray matter volume, cortical thickness, and integrity of white matter tracts in grandiose narcissism, pathological narcissism, and NPD. Also, one of the abovementioned studies on resting-state functional connectivity in grandiose narcissism identified structural correlates (Yang et al., 2015). We will first review results on gray matter, followed by results on white matter.

The first structural imaging study by Schulze and colleagues (2013) investigated gray matter volume in a group of NPD patients compared to matched healthy controls by means of voxel-based morphometry (VBM). Gray matter volume differences were found in the left AI and in the rostral anterior and posterior cingulate cortex, as well as the dorsolateral and mPFC. The region of gray matter volume reduction in the AI overlapped with a region identified as a structural correlate of self-reported empathy in the whole sample (though no significant empathy differences were found in the self-report measure). The other regions also overlap with those commonly implicated in empathy (Lamm et al., 2011), which, taken together, corroborates the notion of reduced empathic functioning in NPD at the level of brain structure. A subsequent study compared gray matter volume in six male NPD patients to controls (Nenadic et al., 2015). Lower gray matter volume was found in the right middle frontal gyrus, left medial prefrontal/anterior cingulate cortex, left middle occipital cortex, left fusiform/inferior temporal cortex, right superior temporal cortex, and in the left lingual gyrus. While some of these areas overlap with those reported by Schulze and colleagues (2013), this study did not find reduced gray matter volume in the AI. A further study investigated structural correlates of pathological narcissism (PNI) in a nonclinical sample: Mao and colleagues (2016) studied cortical thickness and volume using surface-based morphometry in a large sample of young individuals. They found lower cortical thickness and volume in the right dorsolateral prefrontal cortex. Cortical thickness was further negatively associated with pathological narcissism in the right inferior frontal cortex, cortical volume was negatively associated in the right postcentral gyrus and the left mPFC. Finally, the aforementioned resting-state connectivity study by Yang and colleagues (Yang et al., 2015) also reported a positive structural correlation between grandiose narcissism and regional gray matter volume in the right superior parietal lobe, which was evident only in women. The authors interpreted this effect in terms of increased attention seeking in women high in grandiose narcissism, as the superior parietal lobe is part of the dorsal attention network.

Turning now from gray matter to white matter, two studies investigated the integrity of fiber tracts using diffusion-tensor imaging: the aforementioned VBM study by Nenadic and colleagues (2015) additionally investigated fractional anisotropy, a marker of integrity of white matter tracts (Basser et al., 1994), in NPD. Fractional anisotropy was lower in the right frontal lobe the right anterior thalamic radiation, the right anterior temporal lobe, the left anterior/lateral temporal lobe, and the right brain stem. The authors state that “white matter pathology might contribute to the structural cortical deficits [but] obviously, these findings are in need of replication” (p. 185). Such a replication can be seen in the findings of Chester and colleagues (2016), who also investigated the integrity of white matter tracts using fractional anisotropy as an indicator, and grandiose narcissism as a predictor. In a sample of young women and men, the a priori assumed negative relationship of grandiose narcissism and fractional anisotropy in the frontostriatal pathway, connecting the ventral striatum and the medial prefrontal cortex, was confirmed. The authors interpret this finding in terms of a neural disconnect between regions involved in reward (striatal) and intrapersonal (mPFC) processing, which might underlie narcissistic strivings for admiration from the social environment (Back et al., 2013; Morf & Rhodewalt, 2001).

Summarizing the structural findings, the first thing that catches the eye is that all studies find *negative* associations between grandiose narcissism, pathological narcissism, or NPD and different indicators of brain structure or integrity (gray matter volume, cortical volume, and thickness, fractional anisotropy of fiber tracts), with the sole exception of Yang and colleagues (2015), who find a positive association between grandiose narcissism and gray matter volume in women. Though it could be hypothesized that increased self-focus – a core aspect of narcissism – might be accompanied by positive structural correlates in self-related areas such as key nodes of the default mode network, or also the anterior cingulate cortex, this does not seem to be the case, based on the existing evidence. This evidence rather exclusively points to dysfunctional aspects of narcissism in terms of reduced neuronal integrity. To this end, the most consistent finding across the existing studies seems to be lowered gray matter volume in the mPFC (Mao et al., 2016; Nenadic et al., 2015; Schulze et al., 2013).

## 3. Summary and Conclusions

### 3.1 Intrapersonal functions in narcissism

One of the central conclusions that can be drawn from the reviewed neuroscience research on intrapersonal functions in narcissism is that individuals with high levels of grandiose narcissism display exaggerated neurophysiological and neuroendocrine stress reactions to situations involving actual or anticipated ego threat, and also display habitually heightened stress indicators. This could be seen as supporting the long-standing mask model of narcissism, which posits that outward displays of grandiosity serve to compensate for an underlying, fragile, and vulnerable self (Akhtar, 1989). While clinicians working with narcissistic patients widely acknowledge the coexistence of grandiose and vulnerable aspects (Pincus & Lukowitsky, 2010; Ronningstam, 2009), this view is not without controversy in quantitative research on narcissism. Conflicting findings have been obtained for the relationship between explicit and implicit self-esteem in grandiose narcissism, partially supporting (Zeigler-Hill, 2006) or disproving (e.g., Campbell et al., 2007) the mask model view. The neuroscience of narcissism might provide an alternative resolution to this long-standing controversy, as it focuses on observable neurophysiological or neuroendocrine reactions to particular stimuli, or measures established baseline indicators, rather contrasting the global positivity/negativity of implicit or explicit self-concepts all at once, such as, for instance, when comparing measures of implicit and explicit self-esteem. This line of research might allow drawing specific inferences on latent, maybe implicit processes that can be formulated in terms of situational contingencies (cf. Roche et al., 2013; Wright & Edershile, 2018), such as “when confronted with situations that involve actual or anticipated ego threat, individuals with high grandiose narcissism react with increased vigilance and stress responses”. Narcissism, hereby, acts as a moderating factor that can impact either the strength or also the direction of effects. This view is consistent with the threatened egotism model of narcissism (Baumeister et al., 1996; Bushman & Baumeister, 1998), which posits that individuals high in narcissism are highly sensitive to potential ego threat, assign higher self-relevance to potentially threatening situations, and display exaggerated responses facing such threat. Higher subjective self-relevance can also be observed for approach-driven goals (Spencer et al., 2018), as evident in the ergometer study (Woodman et al., 2011).

Interestingly, recent research on communal narcissism (agentic strivings expressed in the communal domain; Gebauer et al., 2012) has unveiled similar discrepancies between behavioral and neurophysiological indicators, as it was found in the ultimatum game that individuals high in communal narcissism are not more likely to reject unfair offers, but display stronger electrophysiological responses (P3 amplitude), indicative of greater emotional sensitivity to these offers (Yang, Sedikides, Gu, Luo, Wang, Yang, et al., 2018). The study of diverging behavioral and neural responses might thus add another layer to understanding the complex relations between self-report, behavior, and involuntary neurophysiological reactions.

Neurophysiological research on the narcissistic self has investigated grandiose narcissism in relation to social rejection and found that imagined or actually experienced rejection goes along with higher systolic blood pressure as a stress indicator (Sommer et al., 2009) and stronger activation in the dACC, AI, and subgenual ACC, regions overlapping largely with the salience network and referred to by the authors as the social pain network (Cascio et al., 2015). These findings complement self-report research on social exclusion in grandiose narcissism, in which individuals high in grandiose narcissism do not report feeling threatened by social exclusion (Besser & Priel, 2010). Neurophysiological research on social exclusion in narcissism thus reveals involuntary stress reactions that might either not be reported, or also not be accessible to the narcissistic individuals themselves. Stronger physiological responses in response to social status threats can already be observed in children (Grapsas et al., 2020). Importantly, these reactions are not without social consequences, as Chester and DeWall (2016) report that those who are habitually high in grandiose narcissism and experience social rejection, as indicated by dACC activity, also display aggressive behavior toward others, as predicted by the threatened egotism model (Bushman & Baumeister, 1998). Similarly, as Lobbestael and colleagues (2014) reported, individuals high in grandiose narcissism have a higher tendency to behave aggressively toward others, which is accompanied by testosterone increase. This shows that a neuroscience approach to understanding narcissism may shed light on mechanisms underlying highly socially relevant phenomena, such as aggressive behavior, which may not be apparent in self-report research. Finally, men high in grandiose narcissism also display exaggerated stress responses as evident in high cortisol levels following performance-related ego threat (as induced by the TSST; Edelstein et al., 2010), which is in line with self-report findings (Besser & Priel, 2010). Whether or not potentially stressful situations induce stress reactions likely depends on the degree of ego threat (in terms of the possibility to fail), as lying about the self is accompanied by effects in the opposite direction (decreases in cortisol, increases in testosterone; Dane et al., 2018). More generally, stimuli that are aversive and self-related, but not self-relevant in any way, such as the loud tones used by Kelsey and colleagues (2001), do not induce stress responses, but rather evoke “cool” and prepared reactions, which reminds of fearlessness traditionally associated with the psychopathy construct (Lykken, 1995; for contemporary model, see Hare & Neumann, 2008).

While the abovementioned experimental paradigms directly challenge the self via exposure to threatening situations, the self-face viewing paradigm used in Jauk, Benedek, and colleagues (2017) cannot be considered intrinsically threatening. Nevertheless, self-face viewing also induced saliency-related activation in the dACC (overlapping with the results reported by Cascio et al., 2015), which indicates that presumably neutral, or potentially even rewarding stimuli can induce higher vigilance to potential ego threat in highly narcissistic men if they are both self-related (in this case also self-referential) and self-relevant (in terms of subjective value). A recent EEG study showed that sensory processing of self-related stimuli is modulated by antagonistic and agentic aspects of narcissism, which further underpins the notion of increased self-relevance for individuals high on narcissism (Mück et al., 2020). Finally, regarding pathological narcissism, which also includes narcissistic vulnerability at an explicit level, even situations that might be expected to induce feelings of power might be perceived as threatening (Stenstrom et al., 2018).

Regarding the interpretation of studies showing activation differences in regions of the salience network – particularly the dACC – for self-relevant stimuli in narcissism, it is important to note that salience network activation cannot per se be attributed to a unitary psychological process (Seeley, 2019). According to the adaptive control hypothesis, the dACC integrates signals from multiple systems under conditions of uncertainty to enable rapid action (Shackman et al., 2011). This adaptive control is likely more relevant facing potential threat than potential reward, also from an evolutionary perspective (de la Vega et al., 2016; Shackman et al., 2011), but the implication of the dACC in processes with positive valence has also been discussed (Shackman et al., 2011). While the observed differences can thus not unequivocally be interpreted in terms of valence, one plausible interpretation of dACC activity in the reviewed studies seems to be that individuals high in narcissism show stronger saliency-related responses facing self-relevant stimuli or situations. On the basis of the understanding of dACC function and the reviewed literature, this seems to be more likely for potentially threatening than rewarding situations, but interpretations might also vary between studies.

The notion of exaggerated physiological reactions in grandiose narcissism is further supported by baseline studies showing that cortisol levels are habitually higher in narcissistic men (Pfattheicher, 2016; Reinhard et al., 2012) and also women who score high on the entitlement/exploitativeness factor of the NPI (Reinhard et al., 2012), which most closely marks the core of the narcissism construct (Krizan & Herlache, 2018; Weiss et al., 2019). Women high in grandiose narcissism also display elevated stress levels (cortisol and alpha-amylase) when negative affect is high (Cheng et al., 2013). Recent research shows that stress levels are even higher on a cellular level in NPD (Lee et al., 2020). Taken together, this is indicative of narcissistic men, and to some extent women, experiencing higher chronic stress levels, presumably due to higher sensitivity to potentially threatening self-relevant situations.

Concerning sex differences in relation to narcissism, available evidence suggests that men display stronger maladaptive reactions than women (Edelstein et al., 2010; Jauk, Benedek, et al., 2017; Reinhard et al., 2012). However, there is also evidence that women display strong reactions when perceived stress is high (Cheng et al., 2013). Sex differences were additionally observed at the level of intrinsic brain network connectivity and might point to different experiential and behavioral tendencies between men and women high in grandiose narcissism (Yang et al., 2015). While some of the reviewed findings support the notion that narcissism might be more maladaptive in men than in women (cf. Jauk, Freudenthaler, et al., 2016; Morf & Rhodewalt, 2001), it is currently unknown whether and to which extent this is due to biological or social factors. Though some research points to associations between sex-specific hormones (testosterone; Noser, 2017; Pfattheicher, 2016) and narcissism, other research also demonstrates the dynamic nature of these associations, which is dependent upon social factors (Dane et al., 2018; Mead et al., 2018). In section 4, we provide suggestions on how future research might address the question of sex and gender differences in neuroscience studies of narcissism.

The notion of an implicit negative self-view in grandiose narcissism receives partial support from structural imaging studies: Chester, Lynam, and colleagues (2016) reported lower integrity of the frontostriatal pathway connecting the ventral striatum and the mPFC. These findings point to a neural disconnect between rewarding and intrapersonal brain areas, which the authors interpret in the way that reduced intrinsically rewarding activity might lead to admiration seeking in the social environment, as posited by self-regulatory models of grandiose narcissism (Back et al., 2013; Morf & Rhodewalt, 2001). This interpretation is consistent with neuroscience research on self-esteem, demonstrating that individuals with high self-esteem display positive rather than negative associations with white matter integrity in the frontostriatal pathway (Chavez & Heatherton, 2017). This might point to a neural dissociation between narcissistically inflated self-esteem and genuinely high self-esteem. This dissociation is particularly interesting given that self-esteem and narcissism are moderately positively related in self-report studies (Campbell, 2001). Neuroscience can thus add a level of analysis that might inform about the underlying psychological processes.

Further structural imaging studies were carried out in NPD patients and groups differing in grandiose and pathological narcissism. The most striking parallel of those studies is that all but one report negative associations between narcissism and different indicators of brain structure; positive associations are hardly evident in the literature. Schulze and colleagues (2013) found lower gray matter volume in the left AI (for a discussion that also includes other personality disorders, see Schulze & Roepke, 2014) and in the anterior and posterior cingulate cortex, as well as the dorsolateral and mPFC. Nenadic and colleagues (2015) found lower gray matter volume in the right middle frontal gyrus, left medial prefrontal/anterior cingulate cortex, left middle occipital cortex, left fusiform/inferior temporal cortex, right superior temporal cortex, and in the left lingual gyrus. Mao and colleagues (2016) found negative relations between pathological narcissism and cortical thickness as well as volume in the right dorsolateral prefrontal cortex. Cortical thickness was further negatively associated with pathological narcissism in the right inferior frontal cortex, cortical volume was negatively associated in the right postcentral gyrus and the left mPFC. Though most of these regions are nonoverlapping, all three studies find effects in the mPFC, which was also implicated in one of the white matter integrity studies discussed above (Chester et al., 2016). The mPFC, as part of the default mode network (Gusnard et al., 2001), is considered central to intrapersonal processing (Wagner et al., 2012), specifically to modulating self-referential processing within the default mode network (Davey et al., 2016). Lower gray matter volume in the mPFC is associated with cumulative lifetime adversity and might reflect general disintegration of intrapersonal functions at a brain structural level (Ansell et al., 2012). These findings are further consistent with multivariate connectivity differences among the mPFC, dACC, and amygdala in grandiose narcissism (Feng et al., 2018). Though the exact nature of these differences is yet to be uncovered, they point to alterations regarding self-referential processes within the default mode network (mPFC), saliency-related processes (dACC), and emotional processes (fear processing in the amygdala; LeDoux, 2000). One structural imaging study, however, did not find differences in the mPFC, but reports a positive association between grandiose narcissism in the right superior parietal lobe for women (Yang et al., 2015). On the basis of the current research, the findings of this study appear hard to reconcile with the rest. The main differences between this study and the others are that it investigated grandiose narcissism (NPI), whereas the other studies investigated either NPD patients or pathological narcissism (PNI), and that structural correlates were studied in interaction with sex.

When considering all of the available evidence of functional, baseline, and structural studies together, the following main points can be summarized from the available evidence:

1. Grandiose narcissism is related to neurophysiological stress reactions indicative of vigilance to actual or anticipated ego threat, which points to vulnerable aspects of grandiose narcissism that are not commonly apparent in self-report research. These reactions manifest in increased salience network activation in the dACC, AI, and partly, also in the ventral/subgenual ACC, as well as increased autonomic (sympathetic; systolic blood pressure) and neuroendocrine (cortisol) indicators. Stressful responses to stimuli occur only for self-relevant material; reactions to aversive stimuli that are self-related but not self-relevant can be “cool”.
2. Grandiose narcissism and NPD are related to altered baseline functioning. This manifests in higher cortisol or 8-OH-DG levels, and distinguishable resting-state connectivity patterns among regions that are also implicated in experimental research (dACC, salience network) as well as the mPFC (default mode network) and the amygdala (limbic system).
3. Grandiose narcissism, pathological narcissism, and NPD are related to brain structural changes, which almost exclusively manifest in negative associations. The most consistent finding is lower cortical volume/gray matter volume in the mPFC for pathological narcissism/NPD. White matter in the frontostriatal pathway, connecting the ventral striatum and the mPFC, displays lower integrity related to narcissistic grandiosity. Prefrontal white matter tracts are also weakened in NPD.

### 3.2 Interpersonal functions in narcissism

The study of interpersonal functions in narcissism mostly targets the long-lasting notion of empathy deficits in narcissism, as included in the diagnostic criteria for NPD since its first mention in the DSM-III (American Psychiatric Association, 1980). While lowered empathic functioning, in terms of reduced sharing of others’ affective states, is well documented for all expressions of narcissism (e.g., Lannin et al., 2014; Pincus et al., 2009; Ritter et al., 2011; Wai & Tiliopoulos, 2012), the exact nature of reduced empathy in narcissism is still a matter of debate. Specifically, it has been questioned whether reduced interpersonal functioning is more indicative of reduced capacity or reduced propensity (Baskin-Sommers et al., 2014), with some evidence pointing into the latter direction (as empathic reactions can be increased by interventions; Hepper et al., 2014). The neuroscience study of narcissism might contribute to an understanding of empathic alterations, as it provides objective assessments of the respective activations complementary to self-report data.

From the literature reviewed above, it can be concluded that individuals with higher levels of pathological and grandiose narcissism generally display lowered activation in brain regions typically implicated in empathizing, particularly the AI, when using high-level social affect paradigms (Fan et al., 2011; Scalabrini et al., 2017). These findings were, on the one hand, explained along the line of greater alexithymia in pathological narcissism (Fan et al., 2011), and on the other hand , as greater self-related attention in grandiose narcissism (Scalabrini et al., 2017).

Regarding the first of these interpretations, alexithymia, in terms of a general incapacity to identify emotions, was also observed to mediate empathy deficits in aggressive offenders (Winter et al., 2017), and is currently being discussed as a general, transdiagnostic functional mechanism of reduced empathy, which could also play a role in borderline personality disorder or psychopathy (Valdespino et al., 2017), depression (Hoffmann et al., 2016), and autism (Bird et al., 2010). With respect to the question of reduced propensity or capacity for empathic reactions in narcissism, the findings of Fan and colleagues (2011) could, tentatively speaking, be seen as more in line with the capacity than the propensity hypothesis (see also Baskin-Sommers et al., 2014). Participants were explicitly instructed to empathize with the faces they saw, and high versus low narcissistic groups gave similar empathy ratings. Yet, they differed in their neural activation, which means that different levels of brain activation were observed under similar conditions. This notion is substantiated by the observation that empathy ratings and activation in the respective neural network are usually positively associated (Kanske et al., 2015). However, alternative explanations cannot be ruled out. For instance, different activation strengths might be accompanied by similar affective experiences (for a parallel from cognitive research, see Neubauer & Fink, 2009), or individuals with higher narcissism levels might provide biased empathy ratings, as this is the case for self-perceptions of general emotional competence (Jauk, Freudenthaler, et al., 2016; Mota et al., 2019). While these interpretative problems are partially epistemological in nature, future research could compare spontaneous and deliberate empathic reactions at a neurophysiological level to further elucidate the nature of reduced empathy in narcissism.

In the second high-level social affect study of narcissism that has been conducted to date, Scalabrini and colleagues (2017) also observed lower activation in the right AI during anticipation of touching a human versus mannequin hand. This is generally in line with the previously discussed findings of Fan and colleagues (2011), as both points to reduced responses in the AI to social stimuli in narcissism. The interpretation, however, differs from the previous one: Scalabrini and colleagues used complemental resting-state scans, which indicated a higher long-range temporal correlation in the AI in individuals higher in grandiose narcissism. The authors interpret this finding in terms of increased self-focus during resting state, particularly with respect to interoceptive and bodily self-aspects. This can be seen as at least partially supporting a hypothesis proposed by Jankowiak-Siuda and Zajkowksi (2013), according to which defective function of the AI, as part of the salience network, leads to a higher likelihood of engaging default mode, self-referential processes in more narcissistic individuals. However, in the study by Scalabrini and colleagues (2017), it was the AI itself, which displayed increased long-range temporal correlation during resting state. Finally, the alexithymia interpretation by Fan and colleagues (2011) and the interoception – interpretation by Scalabrini and colleagues (2017) might be hard to reconcile, as alexithymia and interoceptive abilities are themselves negatively related (Brewer et al., 2016). Thus, for now, it can be concluded that both pathological and grandiose narcissism (grandiose factor of the PNI) are associated with relative deactivation of the AI during high-level social affect paradigms, though the exact nature of this phenomenon must remain subject to future studies.

Turning now to low level, more basic experimental paradigms, two EEG studies found that sensory processing of other related stimuli is not generally impaired in NPD (Marcoux et al., 2014; Zhang et al., 2016). The first of these studies showed that NPD patients even display an increased somatosensory gating effect to observing others’ pain, that is, a stronger reduction of own somatosensory activity (P3 component) when anticipating or observing others’ pain (Marcoux et al., 2014). The second study found no neurophysiological differences in the processing of emotional faces between NPD patients and healthy controls, though the amount of depression reported by NPD negatively modulated P2 amplitudes to neutral and happy faces, which shows that depression amplifies self-focus in NPD (Zhang et al., 2016). Taken together, these studies show that NPD patients do not generally show low-level sensory deficits in the processing of social stimuli, but comorbid symptoms must be considered when studying NPD patients.

Finally, the social–psychological line of neuroscience research on narcissism points to dynamic interactions between narcissism, testosterone, and the subjective experience of power: those with higher baseline testosterone levels were found to score higher on the NPI following a social power manipulation (Mead et al., 2018), which aligns with the finding that – the other way around – testosterone increases in more narcissistic men after acting deceptively (Dane et al., 2018). Testosterone is also correlated with narcissism at baseline in men (Noser, 2017; Pfattheicher, 2016). Taken together, these findings point to the unbowed socially assertive and antagonistic face of narcissism, which likely arises in the absence of a subjective threat to the self.

When considering all of the available evidence together, the following main points can be summarized regarding interpersonal aspects:

1. Individuals with higher levels of pathological and grandiose narcissism display lowered reactions to social stimuli in the AI in high-level paradigms. The AI also shows altered baseline activity in relation to narcissism, and is implicated in intrapersonal processing in narcissism. Though the results seem to be more in line with the notion of a reduced capacity than a reduced propensity for empathic reactions, more research will be needed to elucidate the involved mechanisms.
2. Low-level social information processing, as investigated in electrophysiological studies, is likely not affected by narcissism. However, when studying patients with NPD, comorbid disorders, such as depression, might also alter these processes. This is consistent with the notion that interpersonal functioning in narcissism can substantially vary between different states.
3. The assertive and antagonistic interpersonal style associated with grandiose narcissism is evident in neuroendocrine indicators such as increased testosterone levels. Research on neuroendocrine markers associated with narcissism also points to dynamic situational interactions.

### 3.3 Overall conclusion and conceptual integration

One of the main conclusions that can be drawn from the reviewed research is that intrapersonal functioning in grandiose narcissism is accompanied by heightened vigilance to ego threat and stress responses following subjective ego threat, and also heightened stress indicators at a baseline level (Cascio et al., 2015; Cheng et al., 2013; Edelstein et al., 2010; Jauk, Benedek, et al., 2017; Reinhard et al., 2012; Sommer et al., 2009). These findings are consistent with the threatened egotism model of narcissism (Bushman & Baumeister, 1998) and demonstrate that ego threat may not only have negative consequences for others (in terms of aggressive behavior) but also for the self. Importantly, stress reactions are not necessarily apparent in self-report research and point to vulnerable aspects of grandiose narcissism, which might either not be accessible to, or not reported by individuals high in narcissism. This is in line with behavioral research demonstrating overestimation of emotion-related abilities in narcissism (Ames & Kammrath, 2004; Jauk, Freudenthaler, et al., 2016; John & Robins, 1994; Lobbestael et al., 2016; Mota et al., 2019; Zajenkowski et al., 2018). Also, it is in line with the conclusion of a recent review of stress reactivity in narcissism (Coleman et al., 2019), and shows that it extends to a broader spectrum of research paradigms and methods. The conclusion is also mostly in line with earlier chapters, which could not yet draw on a large amount of studies specific to narcissism (Konrath & Bonadonna, 2014; Krusemark, 2012), and a recent chapter (Krusemark, 2018). In particular, the present review shows that self-relevant stimuli, even when not necessarily stressful, can lead to similar reactions as those that are considered intrinsically stressful or threatening. Individuals high in grandiose narcissism show similar neural reactions in parts of the salience network and also similar “hard wiring” of the frontostriatal pathway as do individuals with low self-esteem (Chavez & Heatherton, 2017; Chester et al., 2016; Jauk, Benedek, et al., 2017; Onoda et al., 2010).

Importantly, self-relevance is a crucial situational variable in this equation, as higher narcissistic individuals do not show stress reactivity (Kelsey et al., 2001; Sylvers et al., 2008), or even cool and prepared reactions facing potentially stress-inducing stimuli which that are not of relevance to the self (Kelsey et al., 2001). Individuals higher in grandiose narcissism also make riskier decisions, accompanied by weaker neurophysiological reactions (Yang, Sedikides, Gu, Luo, Wang, & Cai, 2018), and show reactions indicative of lower stress or even enjoyment following deceptive behavior (Dane et al., 2018). These latter findings might point to common mechanisms underlying certain aspects of narcissism and the psychopathy construct (traditionally characterized by “fearlessness”; Hare & Neumann, 2008).

Interpersonal personality functioning, particularly empathic reactions, is reduced at a behavioral and neurophysiological level when assessed by high-level paradigms and measures (Fan et al., 2011; Scalabrini et al., 2017). More low-level, sensory processing of other related information appears to be intact in NPD (Marcoux et al., 2014; Zhang et al., 2016), in terms of the most extreme expression of grandiose narcissism, though comorbid disorders (depression) might also alter low-level processes (Zhang et al., 2016).

**Figure 2.**
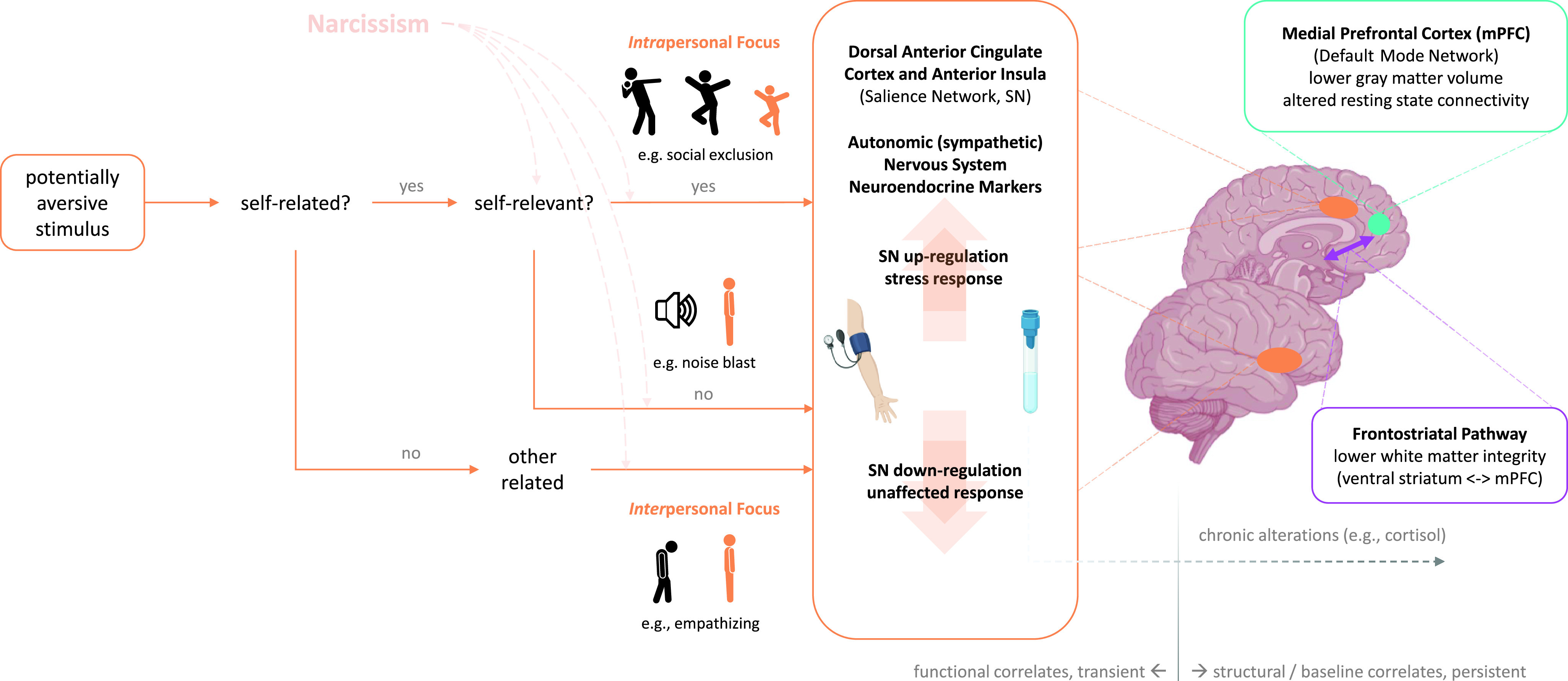
Schematic depiction of central findings of neuroscience studies on narcissism. Dashed lines indicating the moderating influence of narcissism (see section 3) on particular effects. Activation within central nodes of the salience network (orange) and reactions in autonomic (sympathetic) or neuroendocrine markers are higher in situations involving intrapersonal focus when self-relevance is high (potential ego threat). Reactions to aversive self-related stimuli of low self-relevance can be “cool”. Situations involving interpersonal focus (empathy, social touch) lead to down-regulations. At a brain structural and baseline function level, narcissism is related to alterations in regions of the default mode network (cyan) and in the frontostriatal pathway (purple) as well as neuroendocrine markers.

Interestingly, intra- and interpersonal functioning in narcissism partially draw on similar neural networks, as evident in functional, baseline, and structural imaging studies (see Figure 2). The dACC, as part of the salience network (Bressler & Menon, 2010), shows relatively *higher* activation in intrapersonal processing of actual or anticipated ego threat in individuals with higher grandiose narcissism (Cascio et al., 2015; Jauk, Benedek, et al., 2017). The AI, which is also considered as a part of the salience network (Bressler & Menon, 2010) and implicated in empathic processes (Lamm et al., 2011), shows relatively *lower* activation during interpersonal paradigms in highly grandiose and pathologically narcissistic individuals (Fan et al., 2011; Scalabrini et al., 2017), and it shows altered baseline functioning in individuals higher in grandiose narcissism (Scalabrini et al., 2017). Taken together, a very outright interpretation of these findings could be that processing of intrapersonal stimuli in narcissism is accompanied by increased saliency, particularly when these are self-relevant and thus go along with potential ego threat. Processing of other related, interpersonal stimuli, however, seems to be associated with decreased saliency. Activation differences within the salience network thus point to a double dissociation regarding the expression of narcissism and self- and other relatedness of the situation (see Figure 2). This interpretation is partially consistent with a hypothesis proposed by Jankowiak-Siuda and Zajkowski (2013), who also ascribed the salience network a central role in relation to narcissism (though other predictions cannot be evaluated on the basis of the present research), and with a conceptual article pointing to the associations among AI function, narcissism, empathy, and general emotional awareness (i.e., alexithymia; George & Short, 2018), which could also be seen as one potential underlying mechanism (see above; Valdespino et al., 2017).

Interestingly, the dACC was also among the key nodes found to display altered patterns of resting-state connectivity in individuals with higher levels of grandiose narcissism (Feng et al., 2018). Among the other nodes in this study was also the dorsal and ventral mPFC (see Figure 2). The mPFC displayed lower gray matter volume in three out of the four morphometric studies (NPD and pathological narcissism), and is connected to the ventral striatum via the frontostriatal pathway (see Figure 2), which showed decreased white matter integrity in relation to grandiose narcissism (Chester et al., 2016). The mPFC is considered as a part of the default mode network (Gusnard et al., 2001) and central to intrapersonal processing (Davey et al., 2016; Wagner et al., 2012). Lower mPFC volume could indicate a general disintegration of intrapersonal functions, as it is also related to cumulative lifetime adversity (Ansell et al., 2012). Lower white matter integrity between the ventral striatum and the mPFC could further indicate a neural disconnect between regions associated with reward and self-processing (Chester et al., 2016).

Taken together, neuroimaging findings point to functional and structural alterations of regions within the salience network (dACC, AI) and the default mode network (mPFC). Activity within the salience network points to a double dissociation of narcissism and self- versus other related processes.

Some of the conclusions presented here for intra- and interpersonal functioning in narcissism have, from different perspectives or with different emphases, also been expressed in previous integrative works. While we cannot reiterate all points addressed in previous reviews here, we want to highlight some central agreements with recent works: Coleman and colleagues (2019) have recently reviewed studies investigating stress reactivity (psychological, biological, and behavioral) in narcissism, concluding that grandiosity, as well as vulnerability, are generally associated with increased stress reactivity. Krusemark (2018) came to a similar conclusion, stating that “grandiose narcissism involves cardiovascular reactivity in response to aversive stimuli and achievement-related stressors” and “vulnerable narcissism and exploitativeness/entitlement are generally associated with heightened cardiovascular reactivity to interpersonal rejection” (2018, p. 213). However, Coleman and colleagues further argue that “considerable nuance in these associations appears to exist (e.g., what contexts/stressors) […], and under certain stressful conditions narcissism (particularly grandiosity) may confer some level of resilience” (2019, p. 61). We here refer to these moderating factors as *self-relatedness* and *self-relevance*, putting forward the hypothesis that increased salience network up-regulation or stress responses are only observed when both conditions are met. Potentially aversive stimuli or situations that are self-related, but not self-relevant might lead to unaffected responses, or as Coleman and colleagues (2019) further assert, individuals high in grandiosity might have advantages in such situations. Similarly, Krusemark referred to these unaffected responses as a “hypo-reactive physiological profile” (2018, p. 215). Importantly, given that each of us experiences potentially ego-threatening situations, and – as we have argued – individuals high in narcissism might be even more prone to assign self-relevance to such situations, we hypothesize that this might be evident in chronic alterations of neuroendocrine systems or also brain structure in individuals high in narcissism. Krusemark (2018) also asserts that increased physiological reactivity associated with narcissism is accompanied by a heightened risk for cardiovascular disease, and similarly, Coleman and colleagues state that “maladaptive stress-reactivity, particularly when repeated often and over long periods, may cause wear-and-tear on bodily systems that ultimately hasten disease onset and progression” (2019, p. 67). In addition to that, as Konrath and Bonadonna (2014) further note, narcissism is a “risk-positive personality style” (such as, for instance, also evident in the gambling study reviewed here; Yang, Sedikides, Gu, Luo, Wang, & Cai, 2018), which is accompanied by a broad variety of potentially self-defeating, health-relevant behaviors such as sexual risk-taking, substance use, or risky driving. Thus, in addition to physical health risks imposed by increased stress reactivity, narcissism is also associated with a variety of lifestyle factors that might lead to serious health risks over the lifespan (Konrath & Bonadonna, 2014).

Regarding interpersonal functioning in narcissism, George and Short (2018) as well as Di Sarno and colleagues (2018) recently highlighted the role of the AI regarding empathic alterations in NPD. George and Short (2018) further pinpoint that the AI is also involved in general emotional awareness, making alexithymia a possible common mechanism of self- and other related emotional deficits in narcissism. As discussed earlier (see section 3.2), while the AI **–** and more generally the salience network **–** might play a key role in self- versus other related processing in narcissism, the alexithymia interpretation (also originally put forward by Fan et al., 2011) might be hard to reconcile with the notion of increased interoception (Scalabrini et al., 2017), which is why the exact mechanisms of emotion-related alterations in narcissism must remain subject to future study.

Given these areas of overlap with related works, we think that the main contribution of our review is to integrate the available neuroscience literature on narcissism in a theoretical model spanning a broad variety of relevant psychological processes as well as a broad variety of indicators used in neuroscience research. Though such a model cannot adequately depict every finding obtained so far, it is an attempt to identify key topics which are evident across a wide range of studies and previous theoretical works, condense them into testable hypotheses, and put them into a larger perspective, thus moving one step beyond approaches which are either confined to a specific psychological process or a specific set of research methods. Whether or not the assumptions expressed in this model will hold true, we think that providing testable hypotheses is a crucial next step for future neuroscience works on narcissism, which we hope our work does encourage (see following section 4).

## 4. Future Directions

Though existing neuroscience research could already contribute significantly to the understanding of narcissism, there are several ways in which future studies could corroborate and extend the present findings. Here, we provide suggestions on how this might be accomplished:

1. Our main suggestion for future research is to consider the specificity of findings within the multifaceted narcissism construct, which is why we recommend using measures of grandiosity *and* vulnerability when assessing narcissism. This can either be achieved by using separate scales, such as the long-standing standard measures NPI and HSNS, or by using more recently developed composite measures, such as the Five-Factor Narcissism Inventory (FFNI), which allows for a comprehensive assessment of different aspects of narcissism grounded in the Five-Factor Model dimensions (Glover et al., 2012). The recent EEG study by Mück and colleagues (Mück et al., 2020) exemplifies the necessity of considering these, as opposing results were observed for agentic and antagonistic narcissism. For researchers who seek guidance in the choice of scales, Krizan and Herlache (2018) provide an in-depth discussion of the available inventories. In most measures, grandiosity and vulnerability are essentially unrelated in the general population, which easily allows for the sampling of orthogonal factors or groups. Of note, some research suggests that the correlation between grandiosity and vulnerability increases toward the upper end of the grandiosity distribution, which was observed for the NPI/HSNS (Jauk, Weigle, et al., 2017) as well as for the FFNI (Jauk & Kaufman, 2018). This implies that extreme group comparisons, such as performed in some of the reviewed works (Fan et al., 2011; Jauk, Benedek, et al., 2017; Kelsey, Ornduff, McCann & Reiff, 2001; Krusemark, 2009), might lead to a confounding of grandiose and vulnerable aspects. Similar, yet even more intense confounding might arise when studying NPD patients, who frequently have comorbid disorders such as depression (Vater et al., 2013; Zhang et al., 2016). Also, when using the PNI as a measure of pathological narcissism in neuroscience research, one should bear in mind that the close conceptual relation of grandiosity and vulnerability in this construct goes along with high empirical correlations of the respective factors (cf. Wright, Lukowitsky, Pincus, & Conroy, 2010), which might not allow to disentangle their specific effects unless using very large samples. Finally, given potential state fluctuations in narcissism, future neuroscience research could also use momentary assessments of grandiosity and vulnerability, either in the lab or in everyday life.
2. Our second suggestion for future research is to consider the specificity of findings with respect to neighboring personality constructs such as psychopathy or antisocial personality, Machiavellianism, borderline personality, or histrionic personality. More generally speaking, a task for future research in the field might be to investigate whether the effects observed for narcissism are really specific to this particular personality construct, or might also be explained along the lines of general impairments in personality functioning. To date, only a few among the neuroscience studies of narcissism assessed related personality dimensions (Dane et al., 2018; Pfattheicher, 2016; Sylvers et al., 2008) or disorders (Marcoux et al., 2014; Schulze et al., 2013; Wang et al., 2006; Zhang et al., 2016) to probe the specificity of the findings. It might be the case that particularly those findings which cannot unequivocally be related to a specifically narcissistic self-regulatory or interpersonal mechanism, such as baseline alterations of neuroendocrine markers or measures of brain structure, might rather be indicative of general impairments in personality functioning than narcissism per se.
3. Regarding sex differences that are evident in some of the reviewed studies (Edelstein et al., 2010; Jauk, Benedek, et al., 2017; Reinhard et al., 2012; Yang et al., 2015), future research will need to replicate and extend these findings. This concerns on the one hand their consistency, and on the other hand, the extent to which differences are associated with biological sex or social gender. Though some of the findings reviewed here suggest that biological mechanisms might be involved (Noser, 2017; Pfattheicher, 2016), others show that these associations also depend upon social factors (Dane et al., 2018; Mead et al., 2018). Thus, the social component in terms of gender role orientation must not be missed, and only studies assessing both dimensions can inform about their relative significance.
4. The study of interpersonal personality functioning in narcissism could systematically investigate the extent to which social affect and cognition, particularly empathy and perspective-taking, which are increasingly recognized as two different mental processes with different neural correlates (Kanske et al., 2016, 2015), are reduced or impaired in grandiose and vulnerable narcissism. This could be accomplished by contrasting behavioral ratings of empathy and performance measures of perspective-taking with activation in the respective neural networks (see sections 2.1 and 3.2), which might provide some hints on the question of whether reductions in interpersonal functioning are more a matter of reduced propensity or capacity. A possible next step could then be to extend this line of research by considering differences between spontaneous and deliberate social–affective and cognitive processes, which could be accomplished by a variation of test instructions, for instance.
5. Though this might apply to many fields of neuroscience investigations, it needs to be stated that currently, experimental and also baseline and brain structural studies on narcissism used nearly as many different research paradigms or methods as there are studies. To obtain dependable knowledge, future research should thus perform replication and extension studies using established paradigms and research methods. From the present perspective, particularly fruitful next steps would be to systematically disentangle the effects of grandiosity and vulnerability as well as their constituent Five-Factor Model dimensions (see the first point) on well-established ego threat paradigms, such as, for instance, the TSST or the Cyberball paradigm, and well-established social affect and cognition paradigms. Quasi-experimental studies using measures of brain structure or baseline functions could contribute most effectively to acquiring a robust base of knowledge by using data analysis methods corresponding exactly to those of previous studies (at least complemental to new methods) in large datasets. While such studies have recently been carried out for the Five-Factor Model dimensions and yield a rather pessimistic picture regarding the consistency of personality neuroscience findings (Avinun et al., 2020; Valk et al., 2019), it might be that narcissism, as a more specific personality construct, might still display consistent correlates.
6. Finally, it needs to be stated that many neuroscience studies of narcissism used small or very small samples to investigate genuinely subtle associations between personality and physiological or biological indicators, which sometimes include complex relationships such as moderation. This may naturally raise doubt about the robustness and replicability of the reported findings, and we think that such doubt is vital for state-of-the-art planning of future research. However, despite the use of small samples – which is a common problem particularly in earlier neuroscience work – the relatively high degree of consistency across different studies still allows to deduce testable hypotheses, which can inform future confirmatory research using state-of-the-art designs. The model displayed in Figure 2 is an attempt to provide such hypotheses based on the available literature, and we hope it will stimulate future research, which is in line with current methodological standards.

## Footnotes

1 “Empathy” is used for both, social–affective and social–cognitive functions in the AMPD. We will differentiate these functions below.

2 Note, however, that the salience network as such likely does not have a single unitary function (Seeley, 2019), and the results reported here can only be interpreted in the particular context. Please see sections 2.3.1 and 3.1 for a more in-depth discussion.

3 However, the grandiosity factor of the PNI comprises the conceptually related s*elf-sacrificing self-enhancement* scale (see section 1.3).

4 Scalabrini et al. (2017) used experimental and baseline measures; Yang et al. (2015) used structural and baseline measures.

5 One more study used the PNI, but only its grandiose subscale (Scalabrini et al., 2017).

6 For a comprehensive discussion of both components, see Mück et al., 2020.

7 Diagnostic criteria in the categorical system have remained unchanged from DSM-IV to DSM-5.

8 In the second experiment, which was part of this study, the same participants saw affective picture slides. Here, an unexpected positive finding emerged, as those higher in grandiose narcissism showed stronger negative emotional reactions (as evident in negative relations with respiratory sinus arrhythmia and pre-ejection period shortening) toward happy pictures, which the authors interpreted as signs of negative reactions or envy toward others’ happiness. Subjective measures of momentary affect were not assessed in this study.

9 We classified this study as mainly *intra*personal in nature, as participants were videotaped during lying, and not supposed to engage social–affective and cognitive capabilities toward a real other person. However, given that the task likely involves anticipation of imagined others’ reactions, this study could also fit in the interpersonal category.

10 The NI comprises the four dimensions: (1) threatened self, (2) “classical” narcissistic self, (3) idealist self, and (4) hypochondric self, each with several subscales. Though convergent validity information between the NI and scales that are more widely used in psychological research (e.g., NPI, HSNS, PNI) is relatively sparse, the scale descriptions and correlations suggest that the NI measures pathological narcissism, encompassing both aspects of grandiosity (mean correlation with NPI: *r* = .35; Rohmann, Hanke, & Bierhoff, 2019) and, to a somewhat stronger extent, vulnerability (correlation with HSNS: *r* = .54; ibid.).

11 Note that the PNI grandiosity subscale likely encompasses more vulnerable aspects than other measures of grandiose narcissism, such as for instance, the NPI, as “structural analyses suggest that even the purported grandiose aspects are closer to the entitlement-vulnerability side of the spectrum” (Krizan & Herlache, 2018, p. 18). See also section 4.

12 We categorized this study as baseline as the experimental paradigm employed low-level, non-self-related, and non-social auditory stimuli and no voluntary response was required from participants.
